# HPG-Dependent Peri-Pubertal Regulation of Adult Neurogenesis in Mice

**DOI:** 10.3389/fnana.2020.584493

**Published:** 2020-11-27

**Authors:** Sara Trova, Serena Bovetti, Giuliana Pellegrino, Sara Bonzano, Paolo Giacobini, Paolo Peretto

**Affiliations:** ^1^Department of Life Sciences and Systems Biology, Neuroscience Institute Cavalieri Ottolenghi, University of Torino, Orbassano, Italy; ^2^Univ.Lille, Inserm, CHU Lille, U1172 - LilNCog - Lille Neuroscience and Cognition, Laboratory of the Development and Plasticity of Neuroendocrine Brain, Lille, France

**Keywords:** adult neurogenesis, puberty, hypothalamus-pituitary-gonadal axis, GnRH, subventricular zone, dentate gyrus, main olfactory bulb, accessory olfactory bulb

## Abstract

Adult neurogenesis, a striking form of neural plasticity, is involved in the modulation of social stimuli driving reproduction. Previous studies on adult neurogenesis have shown that this process is significantly modulated around puberty in female mice. Puberty is a critical developmental period triggered by increased secretion of the gonadotropin releasing hormone (GnRH), which controls the activity of the hypothalamic-pituitary-gonadal axis (HPG). Secretion of HPG-axis factors at puberty participates to the refinement of neural circuits that govern reproduction. Here, by exploiting a transgenic GnRH deficient mouse model, that progressively loses GnRH expression during postnatal development (*GnRH::Cre;Dicer*^*loxP*/*loxP*^ *mice*), we found that a postnatally-acquired dysfunction in the GnRH system affects adult neurogenesis selectively in the subventricular-zone neurogenic niche in a sexually dimorphic way. Moreover, by examining adult females ovariectomized before the onset of puberty, we provide important evidence that, among the HPG-axis secreting factors, the circulating levels of gonadal hormones during pre-/peri-pubertal life contribute to set-up the proper adult subventricular zone-olfactory bulb neurogenic system.

## Introduction

The reproductive behavior of mammals is orchestrated by a hardwired neuroendocrine network that is greatly influenced in its sexually dimorphic organization and activation by the circulating levels of sex hormones (Nordeen et al., 1985; Romeo, 2003; Arnold, 2009). Starting from early postnatal life the release of sex hormones is under the control of one core part of this system, the hypothalamic-pituitary-gonadal axis (HPG), which includes the gonadotropin-releasing hormone (GnRH) secreting neurons. The GnRH neurons, in turn, integrate external and internal cues perceived by sensory pathways (e.g., olfactory system) characterized by high level of neural plasticity (Lledo and Gheusi, 2003; Boehm et al., 2005; Yoon et al., 2005; Dulac and Wagner, 2006; Oboti et al., 2011; Roa, 2013). This functional organization most likely allows the internal state- and experience-dependent modulation of reproductive behavior. According to this idea, over the last decade several studies in rodents (Mak et al., 2007; Larsen et al., 2008; Oboti et al., 2011, 2017; Larsen and Grattan, 2012; Schellino et al., 2016) have shown that specific reproductive behaviors require a reciprocal crosstalk between the HPG circuits/factors (e.g., gonadal hormones and gonadotropins) and adult neurogenesis (AN) (Mak et al., 2007; Oboti et al., 2011; Brus et al., 2016; Schellino et al., 2016), a striking form of neural plasticity that involves genesis and integration of new neurons during adulthood (Altman and Das, 1965; Alvarez-Buylla and Nottebohm, 1988: Alvarez-Buylla and Garcia-Verdugo, 2002). In mammals, AN occurs constitutively, although at different rates throughout life and species (Lledo and Valley, 2016; Kuhn et al., 2018), in two key sensory regions: the olfactory bulbs (main -MOB- and accessory -AOB-) and the dentate gyrus (DG) of the hippocampus (Bonfanti et al., 1997; Peretto et al., 2001; Alvarez-Buylla and Garcia-Verdugo, 2002; Ming and Song, 2005; Lledo et al., 2006; Oboti et al., 2009, 2011; Gheusi et al., 2013). In adult mice, pheromones, chemosensory cues known to trigger multiple social behaviors and the release of HPG-axis secretory factors (Liberles, 2014), modulate adult neurogenesis in both OB and DG (Mak et al., 2007; Larsen et al., 2008; Oboti et al., 2009, 2011; Feierstein et al., 2010) depending on sex, age, individual experience and internal state of donor and receiver (Mak et al., 2007; Oboti et al., 2009, 2011, 2017; Schellino et al., 2016). In turn, HPG-axis sexual hormones modulate AN based on the same above mentioned factors (Galea and McEwen, 1999; Galea, 2008; Galea et al., 2013; Zhang et al., 2013, 2016). Thus, it is very likely that behaviors elicited by pheromones arise from an interplay/balance between AN and reproductive hormones. Here, to get information about this mechanism, we focused on puberty. Puberty is a critical developmental period characterized by a profound sex-dependent functional reorganization and activation of brain and neuroendocrine circuits underlying reproduction. The onset of puberty in mammals is triggered by increase GnRH-dependent activity of the HPG axis secretion (Romeo, 2003; Sisk and Zehr, 2005; Blakemore et al., 2010; Roa, 2013; Piekarski et al., 2017). Notably, we have previously shown that AN in the AOB of female mice drastically decreases just around puberty and in parallel starts to be modulated by exposure to male pheromones (Oboti et al., 2017), supporting that pubertal brain reorganization also involves a set-up of the AN process. To explore whether this is the case, we took advantage of a mouse model of impaired HPG-axis function, the *GnRH::cre;Dicer*^*loxP*/*loxP*^ (Messina et al., 2016). In these animals Dicer, an RNAse-III endonuclease essential for miRNA biogenesis (Bernstein et al., 2001), is selectively inactivated in GnRH neurons resulting in absence of puberty, and severe hypogonadism and sterility in adulthood caused by persistent GnRH deficiency (Messina et al., 2016). Notably, in these mice impaired gonadotropins release occurs progressively during the infantile period, thus affecting only the onset of puberty (Messina et al., 2016) without altering the critical perinatal endocrine-dependent organizational phase of the brain (Bakker, 2003; Poling and Kauffman, 2013). In addition, to test among the HPG-axis secreting factors the specific modulatory role of pre-/peri-pubertal gonadal hormones on adult neurogenesis, we extended our study on adult females gonadectomized prior to puberty. This model does not reduce (rather it increases) the activity of GnRH and gonadotropins secretions (Czieselsky et al., 2016; Dubois et al., 2016), whereas it excludes the activity of gonadal hormones. Together, our data indicate the levels of pre-/peri-pubertal circulating sex hormones are critical to modulate AN in a sexually dimorphic way, thus suggesting the onset of puberty as a critical time window to set-up this process.

## Materials and Methods

### Animals

All animals were group-housed under specific pathogen-free conditions in a temperature-controlled room (21–22°C) with a 12-h light-dark cycle and *ad libitum* access to food and water. *GnRH::cre(Tg(Gnrh1::cre)1Dlc)* and *Dicer*^*loxP*/*loxP*^ transgenic mouse lines were a generous gift of Dr. Catherine Dulac (Howard Hughes Medical Institute, Cambridge MA) and Dr. Brian Harfe (University of Florida, FL), respectively. CD-1 wild-type mice were purchased from Charles River (Italy). Animal studies were approved by the Institutional Ethics Committees for the Care and Use of Experimental Animals of the Universities of Lille (APAFIS#13387–2017122712209790 v9) and Turin (Protocol Number DGSAF0007085-A05/04/2013); all experiments were performed in accordance with the guidelines for animal use specified by the European Union Council Directive of September 22, 2010 (2010/63/EU). The total number of animals used in this study is *n* = 44. The sex and the number of the animals used in each experiment are specified in figure legends. Transgenic mice were genotyped by PCR using primers listed in Supplementary Table 7. *Gnrh::Cre/Dicer*^*loxP*/*loxP*^ male and female mice were generated by breeding heterozygous males *Gnrh::cre;Dicer*^*loxP*/*wt*^ with homozygous *Dicer*^*loxP*/*loxP*^ females (*Gnrh::cre*^−/−^). Homozygous *Dicer*^*loxP*/*loxP*^ littermates (*Gnrh::cre*^−/−^) were used as control animals. Experiments were designed to minimize the number of animals used.

### Ovariectomy

Juvenile (p21) wild-type female mice were deeply anesthetized with a 3:1 solution of ketamine (Ketavet; Gellini, Italy) and xylazine (Rompun; Bayer, Germany). Two small incisions were performed on each side in the abdominal area, one through the skin and then another through the muscle wall, and ovaries were tied off with absorbable surgical thread and removed (Ström et al., 2012). The muscle and skin incisions were then closed using sutures. After surgery, the animals were positioned under a heat lamp and monitored until recovery. Sham- operated juvenile (p21) female mice have been subjected to the same surgical manipulations without removal of the ovaries. Mice were daily monitored and allowed to recover for 1 week before BrdU injection.

### 5-Bromo-2′-deoxyuridine (BrdU) Treatment

To identify newly generated cells in the AOB, MOB, and DG, adult (p60) *GnRH::Cre/Dicer*^*loxP*/*loxP*^, *Dicer*^*loxP*/*loxP*^ male and female mice and juvenile (p28) wild-type female mice were intraperitoneally injected with BrdU in 0.1 M Tris (pH 7.4) twice a day (interval = 8 h, 100 mg/kg body weight) and sacrificed 28 days later to evaluate survival of adult-generated neurons.

### Tissue Preparation and Sectioning

Mice were deeply anesthetized via an intraperitoneal injection of a 3:1 ketamine (Ketavet; Gellini, Italy) and xylazine (Rompun; Bayer, Germany) solution. All the animals were transcardially perfused with a 0.9% saline solution followed by cold 4% formaldehyde (paraformaldehyde diluted in 0.1 M phosphate buffer, PB), pH7.4. The brains were removed from the skull and post-fixed for 4–6 h in 4% formaldehyde at 4°C. Post-fixation was followed by a cryopreservation step with a 30% sucrose solution in 0.1 M PB pH 7.4 at 4°C. The two hemispheres were separated and embedded in OCT (Sakura Finetek, CA, USA), frozen and cryostat sectioned. Free-floating parasagittal and coronal sections (30 μm) were collected in multi-well dishes to provide representative series of the AOB and MOB/SVZ/DG, respectively. The sections were stored at −20°C in an antifreeze solution (30% ethylene glycol, 30% glycerol, 10% PB: 189 mM NaH_2_PO_4_, 192.5 mM NaOH; pH 7.4) until use.

### Immunohistochemistry

Sections were rinsed in PBS and incubated for 48 h at 4°C in primary antibodies diluted in 0.01 M PBS, pH 7.4, 0.5% Triton X-100, and 1% normal sera that matched the host species of the secondary antibodies. The following primary antibodies were used: anti-Ki67, rabbit IgG polyclonal, dilution 1:1,000, Abcam (ab15580); anti-doublecortin (DCX), goat IgG polyclonal, dilution 1:2,000, Santa Cruz Biotechnology (sc-8066); anti-BrdU, rat IgG monoclonal, dilution 1:5,000, AbD serotec, Bio-Rad Laboratories (OBT0030CX) (Liu et al., 2009). For BrdU immunostaining, sections were pre-treated with 2N HCl for 30 min at 37°C for antigen retrieval and neutralized with borate buffer, pH 8.5, for 10 min. For the avidin-biotin peroxidase method, sections were incubated for 1 h at room temperature in a biotinylated secondary antibody (anti-rat IgG; Vector Laboratories) diluted 1:250 in 0.01 M PBS, pH 7.4, followed by incubation with the avidin-biotin-peroxidase complex (Vector Laboratories). To reveal immunoreactivity, we used 0.015% 3,3′ -diaminobenzidine and 0.0024% H2O2 in 0.05 M Tris-HCl, pH 7.6. After adhesion on gelatin-coated glass slides, sections were mounted in DPX (Merck-Millipore, VWR International PBI, Milan, Italy). For Ki67 and DCX immunostaining, after incubation with primary antibodies, sections were incubated with appropriate fluorochrome-conjugated secondary antibodies for 1,5 h at room temperature. Secondary antibodies were used as follow: anti-rabbit 647-conjugated (1:600; Jackson ImmunoResearch) and anti-goat 488-conjugated (1:400; Jackson ImmunoResearch). Sections were then counterstained with the nuclear dye 4′,6-diamidino-2-phenylindole (DAPI) and coverslipped with the anti-fade mounting medium Mowiol (4–88 reagent, Calbiochem 475904).

### Cell Counting

**Figure 1:**
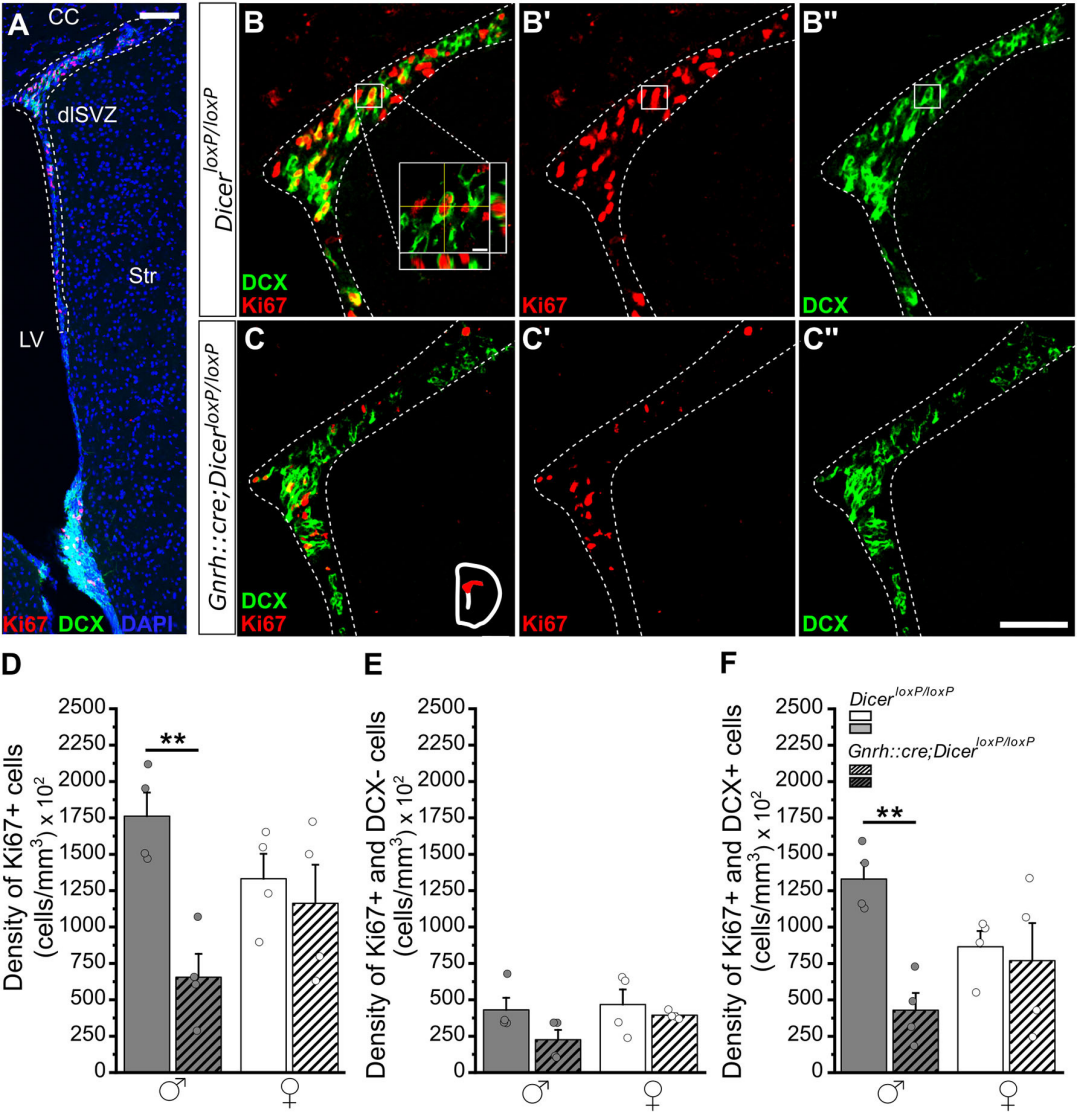
Progenitor proliferation and neuronal differentiation in the dorso-lateral SVZ (dlSVZ) of *GnRH::cre/Dicer*^*loxP*/*loxP*^ and *Dicer*^*loxP*/*loxP*^ mice. **(A)** Representative coronal section of the SVZ, immunolabeled for Ki67 (red), DCX (green), and DAPI (blue) in an adult (P90) *Dicer*^*loxP*/*loxP*^ male mouse. The dotted line indicates the dlSVZ. Scale bar in A = 100 μm. **(B,C)** Immunofluorescence for DCX and Ki67 **(B,C)**, for Ki67 only **(B',C')**, and for DCX only **(B”,C”)** at the level of the dlSVZ in *Dicer*^*loxP*/*loxP*^ **(B–B”)** and *GnRH::cre/Dicer*^*loxP*/*loxP*^ male mice **(C–C”)**. Inset in **(B)** shows a confocal identification of a double-labeled cell (Ki67+/DCX+) including orthogonal planes. Scale bar in inse*t* = 5 μm. Scale bar in C” = 50 μm and applies to **(B,C,B',C',B”)**. **(D–F)** Density of Ki67+ **(D)**, Ki67+/DCX- **(E)**, and Ki67+/DCX+ **(F)** cells in the dlSVZ of *Dicer*^*loxP*/*loxP*^ and *GnRH::cre/Dicer*^*loxP*/*loxP*^ male (*n* = 4 for each genotype) and female mice (*n* = 4 for each genotype). Two-way ANOVA and Tukey's *post-hoc* test, **p = 0.008 in D and **p = 0.009 in F. dlSVZ, dorso-lateral subventricular zone; CC, corpus callosum; Str, striatum; LV, lateral ventricle.

**Figure 2:**
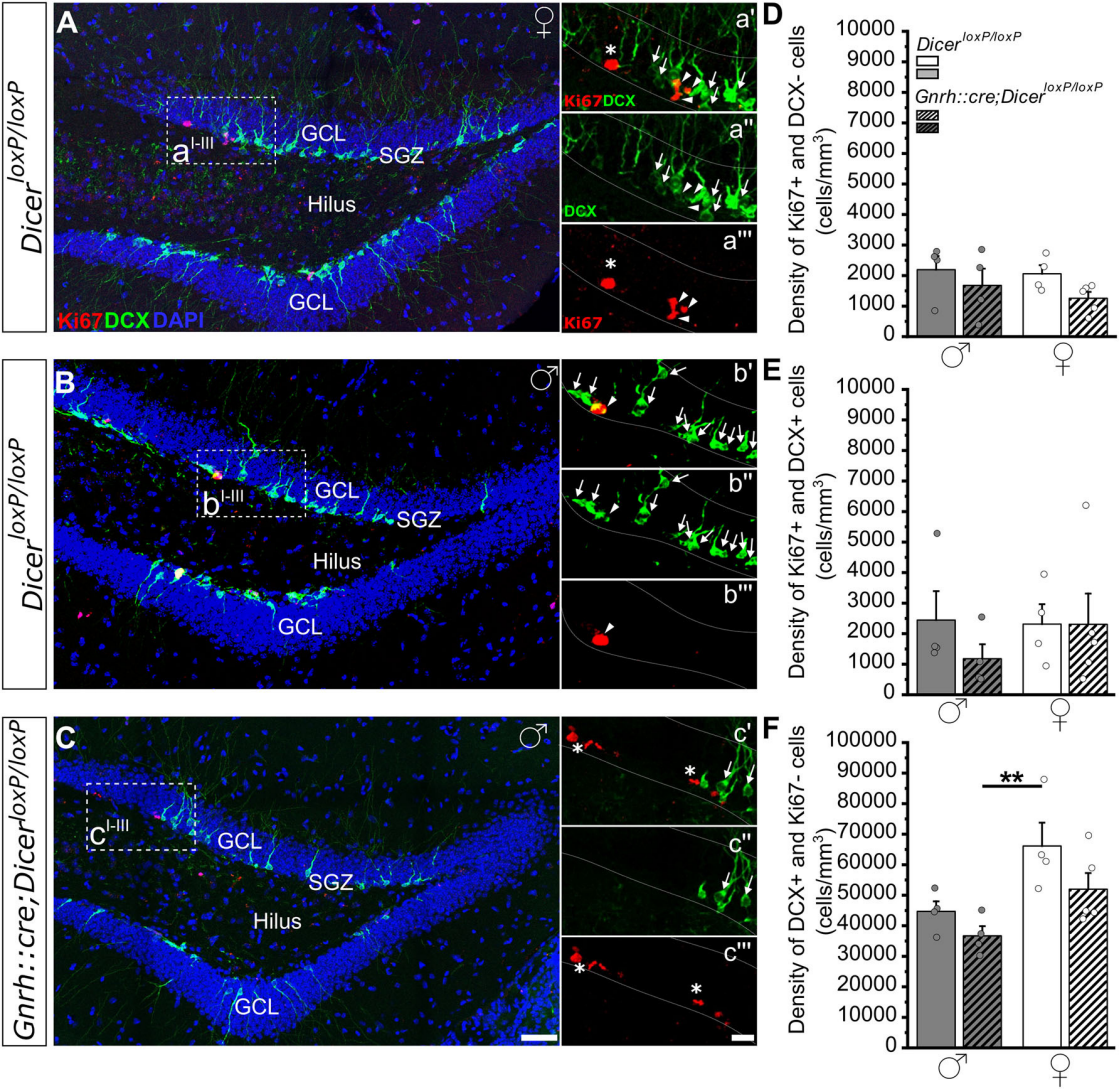
Progenitor proliferation and neuronal differentiation in the DG of *GnRH::cre/Dicer*^*loxP*/*loxP*^ and *Dicer*^*loxP*/*loxP*^ mice. **(A–C)** Representative coronal sections showing the dentate gyrus of the hippocampus in adult (P90) *Dicer*^*loxP*/*loxP*^ female **(A)** and male **(B)** mice, and in a *GnRH::cre/Dicer*^*loxP*/*loxP*^ male mouse **(C)** immunolabeled for Ki67 (red), DCX (green), and DAPI (blue). Insets in **(A–C)** are represented at higher magnification in a'–a”', b'–b”', and c'–c”'. Asterisks in insets indicate Ki67+/DCX- cells, arrows indicate DCX+/Ki67- cells and arrowheads indicate double-labeled Ki67+/DCX+ cells. Scale bar in C = 50μm and applies to **(A,B)**; scale bar in c”' = 10 μm and applies to a'–a”', b'–b”', and c'–c” **(D**–**F)**. Density of Ki67+/DCX- **(D)**, Ki67+/DCX+ **(E)**, and DCX+/Ki67- **(F)** cells in *Dicer*^*loxP*/*loxP*^ and *GnRH::cre/Dicer*^*loxP*/*loxP*^ male (*n* = 4 for each genotype) and female (*n* = 4 control and *n* = 5 *GnRH::cre/Dicer*^*loxP*/*loxP*^) mice. Two-way ANOVA and Tukey's *post-hoc* test. **p = 0.009. GCL, Granule cell layer; SGZ, subgranular zone.

Image acquisition and analysis were performed on either Leica SP5 confocal microscope (Leica Microsystems) or a Nikon microscope coupled with a computer-assisted image analysis system (Neurolucida software, MicroBrightField). For data presented in Figures 1, 2, confocal image z-stacks were captured through the thickness of the slice at 1 μm optical step with 40x objective and three representative coronal sections (30 μm thickness; selected one every 540 μm) per animal were acquired for both the SVZ and DG (for sampling details see Supplementary Figure 1). Z-stacks were imported in NIH Image J software (http://rsb.info.nih.gov/ij/) and analyzed for cell counting. Brightness, color, and contrast were balanced and assembled into panels using Inkscape (Free vector graphics editors). All cell counts were performed blind to the genotype and/or the gender and by taking into account the whole extent of the lateral SVZ (dorso-lateral + ventro-lateral SVZ; Supplementary Figure 1B, left panels) and the hippocampal DG (SGZ+GCL subregions; Supplementary Figure 1B, right panels). The number of Ki67-positive nuclei and double-labeled Ki67 and DCX positive cells was manually counted in the SVZ and in the hippocampal DG. The number of DCX-positive cells was established only in the SGZ/GCL. In each section, the boundaries of the SVZ or of the DG were traced using DAPI staining and areas were automatically calculated using Image J software and multiplied by the thickness of the section (30 μm) to estimate the volume in μm^3^ and finally converted into mm^3^. Cell density was calculated for each section by dividing the total number of labeled cells within the section by the volume of the area of interest (either SVZ or SGZ+GCL) and expressed as number of labeled cells per mm^3^. To estimate the total volume of the lateral SVZ area (comprising the dorso-lateral corner and the whole lateral wall up to the most ventral tip; Supplementary Figure 1C), the area of each section encompassing the SVZ (six to seven sections out of one series per animal, 180 μm intersection intervals; anteroposterior axis: Bregma from +1.3 mm to −0.6 mm) were manually traced based on DAPI staining and automatically calculated by Neurolucida software and the total volumes were estimated by applying the Cavalieri method (Prakash et al., 1994). The same strategy was used for the analysis of the dorsal DG (Supplementary Figure 1D), wherein the area comprising SGZ plus GCL subregions (SGZ+GCL; six to eight sections out of one series per animal, 180 μm intersection intervals; anteroposterior axis: Bregma from −1.3 to −3.3 mm) was manually traced based on DAPI signal and the total volumes were estimated by applying the Cavalieri method (Prakash et al., 1994).

**Figure 3:**
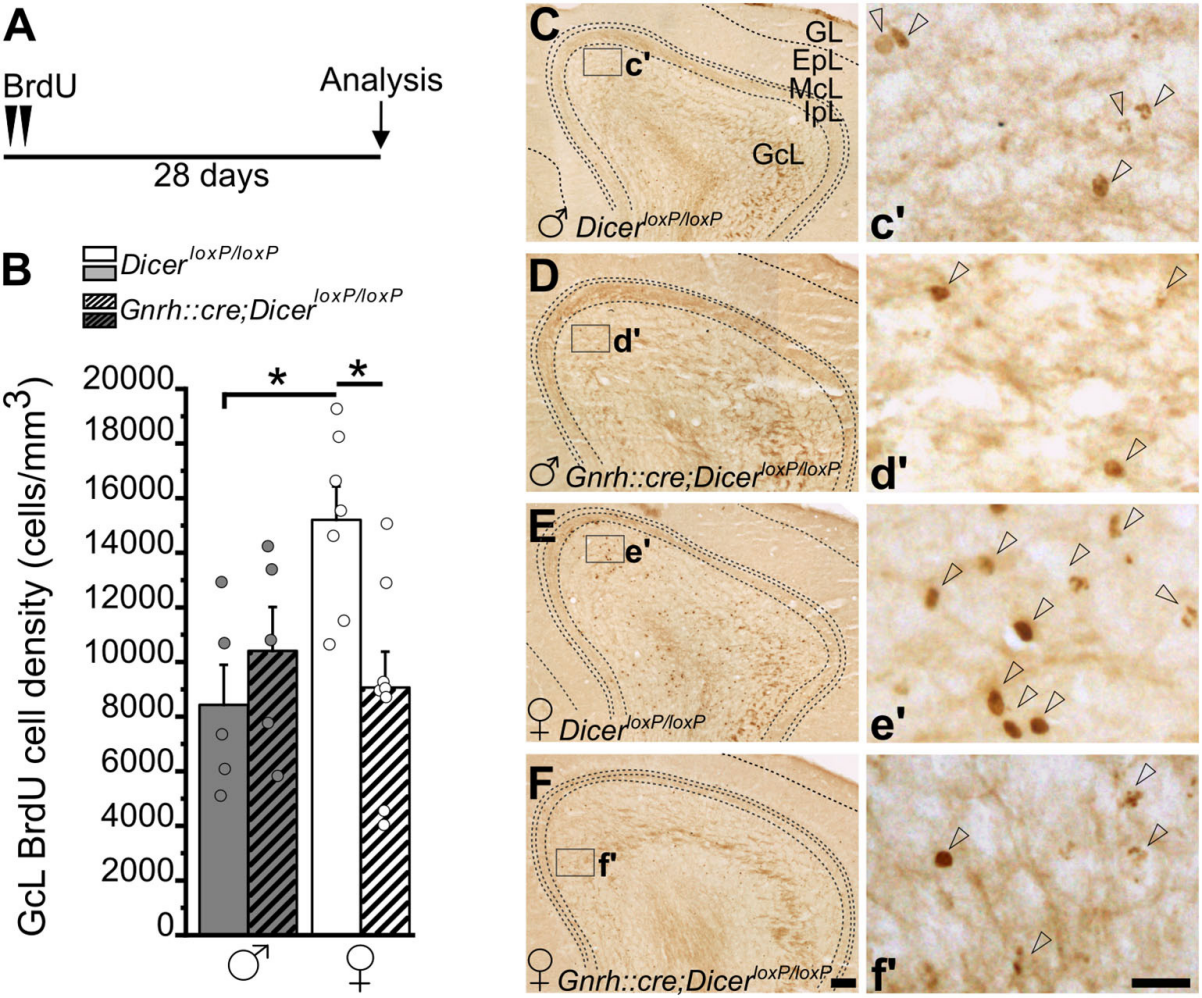
Survival of newborn granule cells in the Main Olfactory Bulb of *GnRH::cre/Dicer*^*loxP*/*loxP*^ and *Dicer*^*loxP*/*loxP*^ mice. **(A)** Experimental protocol. **(B)** BrdU-positive cell density in the Main Olfactory Bulb (MOB) GcL of *Dicer*^*loxP*/*loxP*^ and *GnRH::cre/Dicer*^*loxP*/*loxP*^ male (*n* = 5 for each genotype) and female (*n* = 7 control and *n* = 8 *GnRH::cre/Dicer*^*loxP*/*loxP*^*)* mice. Two-way ANOVA and Tukey's *post-hoc* test, *p = 0.016 control males vs. control females, *p = 0.013 control vs. *GnRH::cre/Dicer*^*loxP*/*loxP*^ females. **(C–F)** Representative images of MOB coronal sections showing BrdU-positive newborn neurons in *Dicer*^*loxP*/*loxP*^ and *GnRH::cre/Dicer*^*loxP*/*loxP*^ male **(C,D)** and female **(E,F)** mice, 28 days after BrdU administration. Insets in **(C–F)** are represented at higher magnification in c'–f'. Arrowheads indicate BrdU-positive nuclei. Scale bar in F = 100 μm and applies to **(C–E)**; scale bar in f' = 10 μm and applies to c'–e'. GL, glomerular layer; EpL, external plexiform layer; McL, mitral cell layer; IpL, Internal plexiform layer; GcL, granule cell layer.

**Figure 4:**
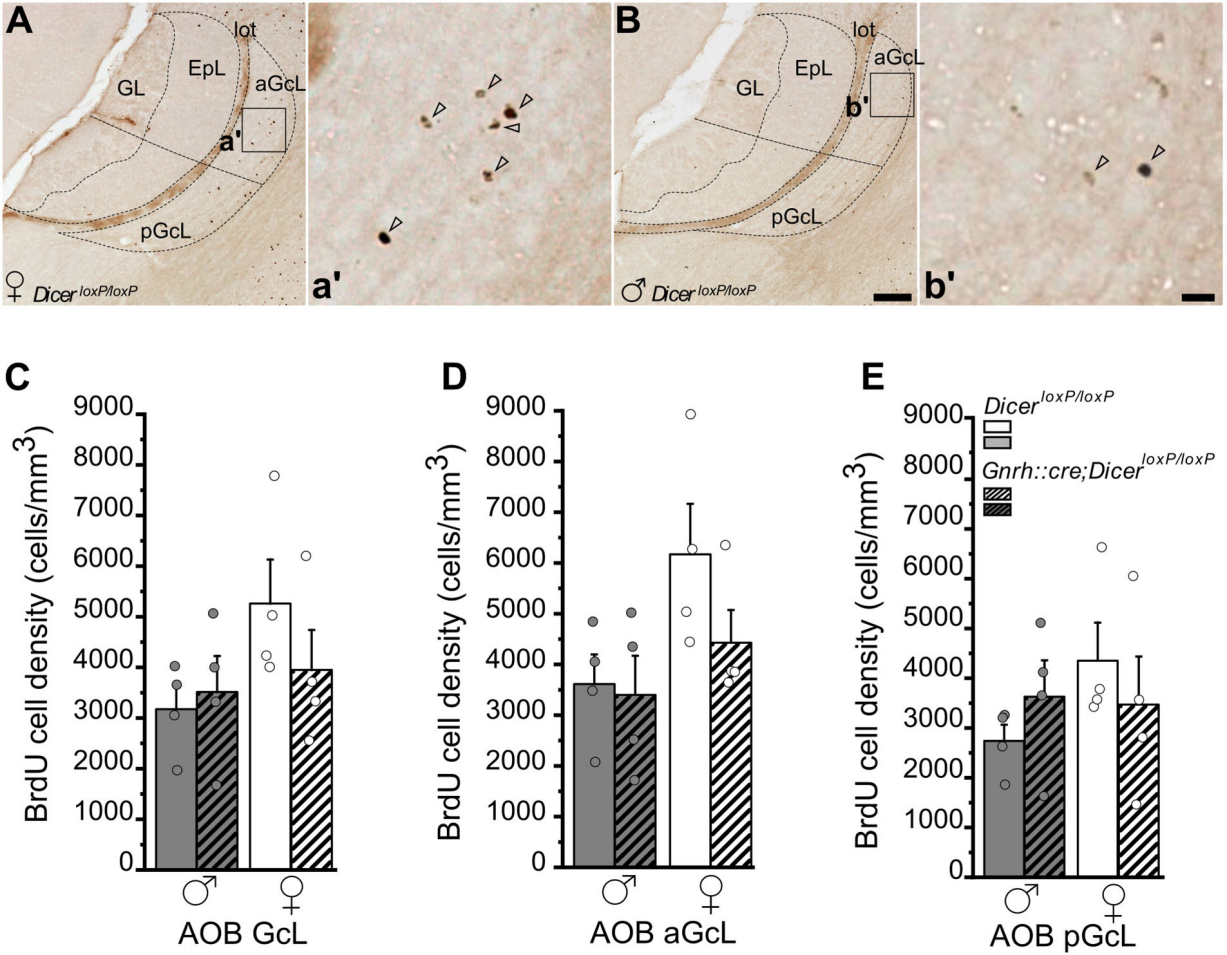
Survival of newborn granule cells in the Accessory Olfactory Bulb of *GnRH::cre/Dicer*^*loxP*/*loxP*^ and *Dicer*^*loxP*/*loxP*^ mice. **(A,B)** Representative images of Accessory Olfactory Bulb (AOB) sagittal sections showing BrdU-positive newborn neurons in *Dicer*^*loxP*/*loxP*^ female **(A)** and male **(B)** mice, 28 days after BrdU administration. Inset in **(A,B)** are represented at higher magnification in a',b'. Arrowheads indicate BrdU-positive nuclei. Scale bar in B = 50 μm and applies to **(A)**; scale bar in b' = 10 μm and applies to a'. **(C–E)** BrdU-positive cell density in the whole AOB-GcL **(C)**, anterior GcL **(D)**, and posterior GcL **(E)** of *Dicer*^*loxP*/*loxP*^ and *GnRH::cre/Dicer*^*loxP*/*loxP*^ male (*n* = 4 for each genotype) and female (*n* = 4 for each genotype) mice. Two-way ANOVA and Tukey's *post-hoc* test. GL, glomerular layer; EpL, external plexiform layer; aGcL, anterior granule cell layer; pGcL, posterior granule cell layer; lot: lateral olfactory tract.

**Figure 5:**
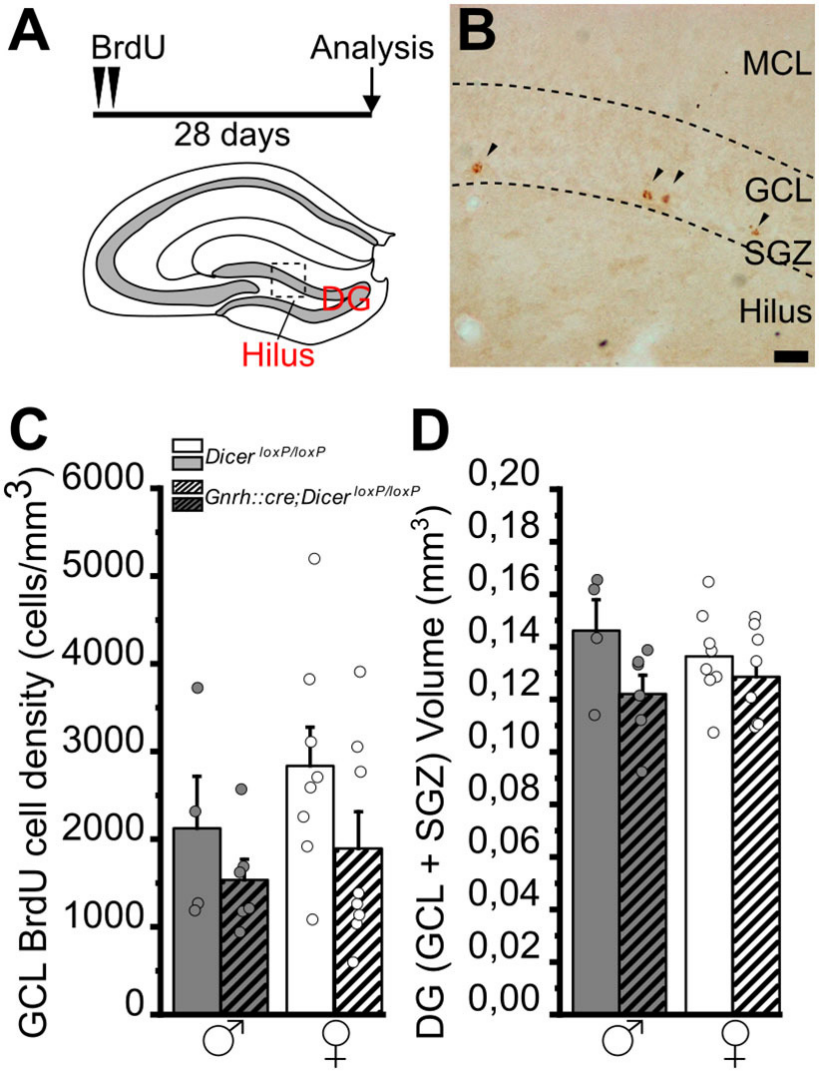
Survival of newborn granule cells in the dentate gyrus of the hippocampus of *GnRH::cre/Dicer*^*loxP*/*loxP*^ and *Dicer*^*loxP*/*loxP*^ mice. **(A)** Experimental protocol (upper) and schematic coronal view of the hippocampus (bottom) of an adult mouse brain indicating the Dentate Gyrus (DG) and its Hilus. **(B)** Representative image of a coronal section of the upper blade of the DG [approximative position inset in **(A)**] showing BrdU labeled nuclei (arrowheads) in a *Dicer*^*loxP*/*loxP*^ female, 28 days after BrdU administration. Scale bar in B = 20 μm. **(C)** BrdU-positive cell density in the GCL+SGZ of the DG in *Dicer*^*loxP*/*loxP*^ and *GnRH::cre/Dicer*^*loxP*/*loxP*^ male (*n* = 4 control and *n* = 6 *GnRH::cre/Dicer*^*loxP*/*loxP*^) and female (*n* = 8 for each genotype) mice. Two-way ANOVA and Tukey's *post-hoc* test. **(D)** Volume of the GCL+SGZ in *Dicer*^*loxP*/*loxP*^ and *GnRH::cre/Dicer*^*loxP*/*loxP*^ male (*n* = 4 control and *n* = 6 *GnRH::cre/Dicer*^*loxP*/*loxP*^) and female (*n* = 8 for each genotype) mice. Two-way ANOVA and Tukey's *post-hoc* test. DG, dentate gyrus; GCL, granule cell layer; MCL, molecular cell layer; SGZ, subgranular zone.

**Figure 6:**
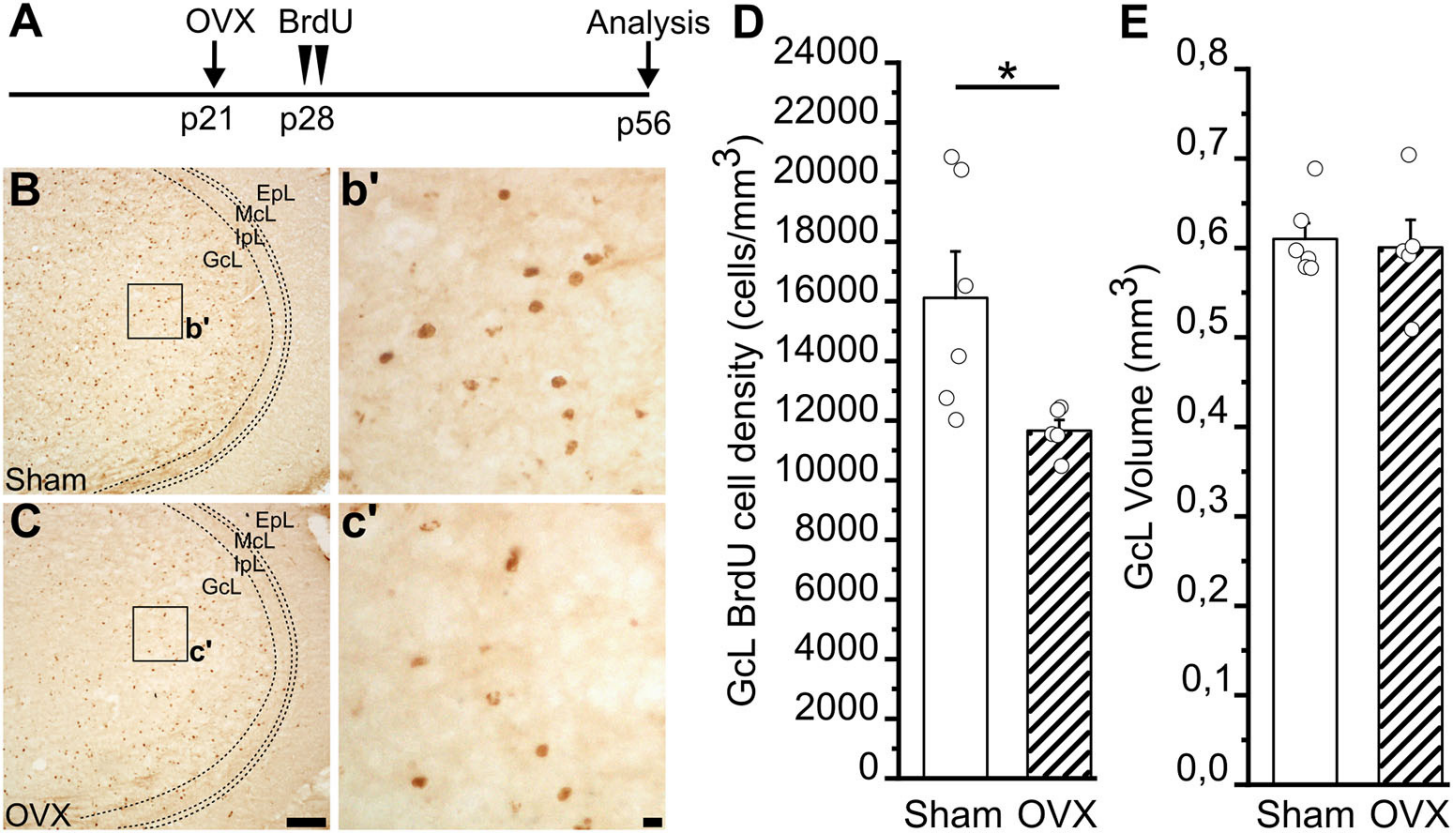
Survival of newborn granule cells in the Main Olfactory Bulb of Sham and ovariectomized female mice. **(A)** Experimental protocol: female mice were ovariectomized at p21 and injected with BrdU 1 week later (p28). Newborn cells were then quantified in the GcL of the main olfactory bulb (MOB) 28 days after BrdU injection (p56). **(B,C)** Representative images of MOB GcL coronal sections showing BrdU-positive newborn neurons in sham **(B)** and ovariectomized, OVX **(C)**, females 28 days after BrdU administration. Scale bar in C = 100 μm and applies to **(B)**; scale bar in c' = 10 μm and applies to b'. **(D)** BrdU-positive cell density in the MOB GcL of sham (*n* = 6) and ovariectomized (*n* = 5) female mice. **(E)** Volume of GcL in sham (*n* = 6) and ovariectomized (*n* = 5) female mice. Student's *t*-test, *p = 0.032.

For data presented in Figures 3–**6**, the number of BrdU-positive nuclei was established using the Neurolucida software (MicroBrightField) by counting peroxidase/DAB-stained nuclei in three representative MOB coronal (anterior, medial, and posterior) sections (30 μm thickness; 180 μm intersection interval) per animal in the granular cell layer (GcL; 40x objective). In each section, the boundaries of the GcL were traced and its area was automatically calculated by Neurolucida software. To identify the anterior and posterior subdivisions of the AOB GcL, we first measured the total length of the AOB glomerular layer and then we traced a line in the exact half of the glomerular layer to define the underlying subdivision of the granular cell layer. This subdivision has been previously described based on Gαi immunohistochemical staining, which selectively labels the anterior half part of the glomerular layer of the AOB (Jia and Halpern, 1996; Dudley and Moss, 1999). Counting was conducted using a systematic random sampling method by overlaying each section with a virtual counting grid (squares size 80 × 80 μm) and counting the number of positive cells in one square of the grid (one of every two) through sequential translation of the counting frame until the area of interest was entirely covered. This procedure allowed us to analyze about one-fourth of the area of interest. Cell density (number of labeled profiles/mm^3^) was calculated by multiplying cells x four and by multiplying the area measurements by the mean section thickness (30 μm) [Σ of sampled areas μm^2^ × 30 μm]. In the AOB, the number of BrdU-positive nuclei was established by counting peroxidase/DAB-stained nuclei in six para-sagittal (representing the entire AOB) sections (30 μm thickness, 60 μm intersection interval) per animal in the granular cell layer. For data presented in **Figure 5**, the number of BrdU-positive nuclei was established by counting peroxidase/DAB-stained nuclei in 8 coronal (representing the entire DG) sections (30 μm thickness, 180 μm intersection interval) per animal in the granular cell layer. Volumes were estimated applying the Cavalieri method (Prakash et al., 1994).

### Statistical Analysis

Values are expressed as mean ± s.e.m unless otherwise stated. A Shapiro-Wilk normality test was run on each experimental sample. When comparing two populations of data, two-tailed Student's *t*-test was used to calculate statistical significance. When more than two populations of data were compared, two-way ANOVA with Tukey's *post-hoc* test was used. Statistical analysis was performed with Origin software (OriginLab). The number of animals and *p*-values are reported in figure legends and in Supplementary Tables 1.1–6.2.

## Results

In order to get new insights into the mechanisms regulating the activity of brain networks underlying reproductive behavior, we investigated the process of adult neurogenesis in the *GnRH::cre;Dicer*^*loxP*/*loxP*^ mice. These animals never go through puberty due to development of a juvenile impaired secretion of GnRH that persists during adulthood, resulting in a chronic deficiency of secretion of gonadotropins and gonadal hormones (Messina et al., 2016). In addition, to distinguish among these factors the relative contribution of gonadal hormones in the peripubertal setting of adult neurogenesis, we examined the survival of newborn neurons in the olfactory bulb of female mice gonadectomized soon before puberty.

### Juvenile Impaired Secretion of GnRH in Male Mice Decreases the Number of Proliferating Neuronal-Committed Progenitors in the Adult Dorso-Lateral SVZ

By combining the expression of Ki67, an endogenous marker of cell proliferation expressed during all active phases of the cell cycle with the exception of G0 phase (Zacchetti et al., 2003), with the doublecortin (DCX), a brain-specific microtubule-associated protein expressed in progenitors fated to the neuronal lineage, neuroblasts, and immature neurons (Gleeson et al., 1998; Francis et al., 1999), we examined the early critical steps of AN process focusing on the proliferative activity of neural progenitors and their commitment toward the neuronal lineage. The analysis was performed in both sexes and adult neurogenic niches of *GnRH::cre;Dicer*^*loxP*/*loxP*^ and *Dicer*^*loxP*/*loxP*^ (control) mice. Specifically, we quantified: (i) the total number of proliferating progenitors (Ki67+ cells); (ii) the proliferating progenitors committed toward the neuronal lineage (Ki67+/DCX+ double-labeled cells); (iii) the proliferating progenitors not expressing the neuronal marker DCX (Ki67+/DCX- cells). Moreover, in the DG of hippocampus we also quantified: (iv) the number of post-mitotic neuroblasts/immature neurons (DCX+/Ki67- cells). This latter cell population was not quantified in the SVZ since a careful examination of single DCX immune-positive cells is not feasible in this region due to its peculiar anatomical organization. Finally, according to the existence of diverse spatio-temporal progenitor microdomains along the rostro-caudal and dorso-ventral axes of SVZ (De Marchis et al., 2007; Merkle et al., 2007, 2014), quantifications in this region were performed by considering the SVZ subdivided into dorso-lateral (dlSVZ) and ventro-lateral (vlSVZ) sub-domains (Figure 1; Supplementary Figures 1, 2).

In the dlSVZ, two-way ANOVA analysis revealed a significant effect of gender x genotype interaction on the density of proliferating progenitors [Ki67+ cells; Overall two-way ANOVA, gender x genotype *F*_(1, 12)_ = 5,795, *p* = 0.033; Supplementary Table 1.1]. A significant decrease in the number of Ki67+ cells emerged in *GnRH::cre;Dicer*^*loxP*/*loxP*^ males compared to control males while no difference was detected among genotypes in females (Tukey's *post-hoc* test, *p* = 0.008 control vs. *GnRH::cre;Dicer*^*loxP*/*loxP*^ males; Figures 1B–D; Supplementary Table 1.1). The quantification of double labeled Ki67+ and DCX+ cell density similarly revealed a significant effect of gender x genotype interaction [Ki67+ and DCX+ cells; Overall two-way ANOVA, gender x genotype *F*_(1, 12)_ = 6,189, *p* = 0.028; Supplementary Table 1.3] and multiple comparison revealed that differences were restricted to male mice. Indeed, the density of Ki67+ and DCX+ cells significantly decreased in *GnRH::cre;Dicer*^*loxP*/*loxP*^ compared to control males (Tukey's *post-hoc* test, *p* = 0.009 control vs. *GnRH::cre;Dicer*^*loxP*/*loxP*^ males; Figures 1B,C,F; Supplementary Table 1.3). No difference was detected in either Ki67+/DCX- cell densities of the dlSVZ (Figure 1E; Supplementary Table 1.2) and among all parameters (gender and genotype) and groups when referred to the vlSVZ subdomain of both male and female mice (Supplementary Figures 2A–F; Supplementary Tables 1.4–1.6). Finally, we evaluated SVZ volume to assess whether the decrease in Ki67+ and Ki67+/DCX+ cell density in *GnRH::cre;Dicer*^*loxP*/*loxP*^ males compared to control males reflected a general alteration of SVZ volume in this group. Overall, two-way ANOVA revealed a significant effect of genotype [genotype *F*_(1, 8)_ = 8,836, *p* = 0.018; Supplementary Table 1.7] on SVZ volume and a significant decrease emerged when *GnRH::cre;Dicer*^*loxP*/*loxP*^ males were compared to control females (Tukey's *post-hoc* test, *p* = 0.042; Supplementary Figure 1C), but not to control males (Supplementary Figure 1C; Supplementary Table 1.7).

In the DG of the hippocampus, no significant difference was found among groups when we examined the densities of either Ki67+ and DCX- cells (Figure 2D; Supplementary Table 2.1) or Ki67+ and DCX+ cells (Figure 2E; Supplementary Table 2.2). By contrast, two-way ANOVA analysis revealed a significant effect of gender on the density of DCX+ and Ki67- cells [Overall two-way ANOVA, gender *F*_(1, 13)_ = 12,139, *p* = 0.004]. The Tukey's *post-hoc* analysis indicated that *GnRH::cre;Dicer*^*loxP*/*loxP*^ male mice show a reduction in the density of DCX+ and Ki67- cells when compared to control females (*p* = 0.009; Figure 2F; Supplementary Table 2.3). No difference in DG volume was detected among groups (Supplementary Figure 1D; Supplementary Table 2.4).

Overall, the above data show that impaired juvenile GnRH secretion leads to long-term alterations of the early steps of AN in a sex-specific way, mostly affecting the process of progenitor differentiation in the dlSVZ of male mice.

### Juvenile Impaired Secretion of GnRH Decreases the Survival of Newborn Neurons in the MOB of Adult Female Mice

Another critical step of the AN process is the selection phase occurring during the integration of newborn neurons into the target circuits (Petreanu and Alvarez-Buylla, 2002; Winner et al., 2002; Alonso et al., 2008; Oboti et al., 2009, 2011). Although the dynamic of this process in the OBs and DG niches occurs with small temporal differences, after 1 month from genesis the large majority of survived newborn neurons is functionally integrated in both regions (Petreanu and Alvarez-Buylla, 2002; Van Praag et al., 2002; Ambrogini et al., 2004; Ge et al., 2007; Oboti et al., 2009, 2011; Yang et al., 2015). Thus, to evaluate whether impaired secretion of GnRH influences newborn neurons survival in the AN target tissues, we labeled a cohort of newly generated neurons through i.p injection of the thymidine analog BrdU (Nowakowski et al., 1989) and quantified their density 28 days later in both the MOB, AOB and DG (Figures 3–**5**, respectively).

Quantification in the MOB (Figure 3) was performed in the granule cell layer (GcL) where the vast majority of adult-generated interneurons integrates (Winner et al., 2002). Two-way ANOVA analysis revealed a significant interaction between gender and genotype [Overall two-way ANOVA, gender x genotype, *F*_(1, 21)_ = 8,160, *p* = 0.009; Supplementary Table 3.1]. The Tukey's *post-hoc* analysis showed a significant reduction of BrdU+ cell density in *GnRH::cre;Dicer*^*loxP*/*loxP*^ vs. control females (Figures 3B,E,F; *p* = 0.013; Supplementary Table 3.1), while no difference was found between *GnRH::cre;Dicer*^*loxP*/*loxP*^ vs. control males (Figures 3B–D; Supplementary Table 3.1). Interestingly enough, sexual dimorphism occurs in control animals, with females showing higher BrdU+ cell density in the MOB GcL compared to males (Figures 3B,C,E; Tukey's *post-hoc* test, *p* = 0.016 control females vs. control males; Supplementary Table 3.1). Importantly, the GcL volume did not change among all experimental groups (Supplementary Figure 3A; Supplementary Table 3.2), further supporting the above results represent group specific variations of the AN process rather than anatomical changes.

In the AOB GcL, analysis of BrdU+ cell density did not indicate any significant difference among groups, although control females showed in average higher values (Figure 4C; not significant; Supplementary Table 4.1). When the AOB was divided into its anterior and posterior functional subdivisions (Figures 4A,B,D,E; Jia and Halpern, 1996; Sugai et al., 1997; Dudley and Moss, 1999; Kumar et al., 1999; Martìnez-Marcos and Halpern, 1999), overall two-way ANOVA analysis revealed a significant effect of gender on BrdU+ cell density in the anterior AOB [Figure 4D; Overall two-way ANOVA, gender *F*_(1, 12)_ = 5,499, *p* = 0.037; Supplementary Table 4.2] although no specific differences were detected by Tukey's *post-hoc* test (Supplementary Table 4.2). No change in the density of BrdU+ cells emerged in the pAOB (Figure 4E; Supplementary Table 4.3) and no difference in the volume of the AOB GcL was detected (Supplementary Figure 3B; Supplementary Table 4.4).

In the DG of the hippocampus, the two-way ANOVA analysis did not show any significant difference of BrdU+ cell density among groups (Figures 5A–C; Supplementary Table 5.1). However, overall two-way ANOVA revealed an effect of genotype on DG volumes [gender *F*_(1, 22)_ = 4,484, *p* = 0.046; Supplementary Table 5.2], although no specific differences were identified by multiple comparison Tukey's *post-hoc* test (Figure 5D; Supplementary Table 5.2).

Overall, the above data regarding newborn cell survival support that impaired juvenile GnRH secretion also triggers long-term alteration in the late stages of the AN process in a sex- and niche-specific way, involving only females and the main olfactory bulb. In addition, considering the effect of genotype on DG volumes, as well as in SVZ volumes (see section Juvenile impaired secretion of GnRH in male mice decreases the number of proliferating neuronal-committed progenitors in the adult dorso-lateral SVZ), we cannot exclude that impaired HPG axis could bring to developmental morphological changes including volume variations in neurogenic niches.

### Pre-pubertal Ovariectomy Affects the Survival of Adult-Born Neurons in the MOB

Impaired juvenile GnRH secretion in *GnRH::cre;Dicer*^*loxP*/*loxP*^ mice does not allow pre-pubertal rise of LH and FSH and in turn of gonadal hormones (Messina et al., 2016). Each one of these factors of the HPG axis can thus contribute to the alterations of the AN process identified in this model. In this context, we addressed the relative contribution of gonadal hormones in females. To this aim, we investigated newborn cell survival in the MOB (altered in the *GnRH::cre;Dicer*^*loxP*/*loxP*^ females) of adult wild-type females gonadectomized just before puberty onset. In this way, the GnRH system and gonadotropins are preserved, but the secretion of ovarian hormones impaired. Wild-type female mice were ovariectomized (OVX) at postnatal day 21 (p21), just before the typical raise of gonadal hormones that drives the puberty onset (Oboti et al., 2017), and injected with BrdU 7 days later, at p28 (Figure 6A). The density of BrdU+ nuclei in the GcL of the MOB was analyzed 28 days after BrdU injection. Notably, we observed a significant reduction of BrdU+ cell density in the GcL of the MOB in the OVX compared to sham-operated group (Student's *t*-test, *p* = 0.032; Figures 6B–D; Supplementary Table 6.1). No difference was found in the MOB GcL volumes between OVX and control animals (Student's *t*-test, *p* = 0.785; Figure 6E; Supplementary Table 6.2). This latter experiment supports an important contribution of gonadal hormones to the alterations of AN process identified in *GnRH::cre;Dicer*^*loxP*/*loxP*^ mouse model (at least for what concerns cell survival in the MOB of adult females).

## Discussion

We have recently shown that neurogenesis in the olfactory bulb of female mice is significantly modulated during puberty (Oboti et al., 2017), a critical stage of life characterized by increase secretion of gonadal hormones (Sisk and Zehr, 2005) and refinement of neural circuits that drive reproduction (Piekarski et al., 2017). This supports the multimodal tuning of sex circuits occurring during peri-pubertal life can also involve a set-up of the AN process, possibly through gonadal hormones or more in general HPG factors/secretions (e.g., GnRH, LH, FSH, gonadal hormones). Accordingly, adult neurogenesis is modulated by sexual hormones (Galea, 2008; Ponti et al., 2018), it becomes sensitive to environmental reproductive cues (i.e., pheromones) after puberty (Oboti et al., 2017), and it is implicated in the control of sex-behaviors in several mammalian species (Galea and McEwen, 1999; Mak et al., 2007; Migaud et al., 2015; Brus et al., 2016; Alvarado-Martínez and Paredes, 2018).

Here, to investigate how the pubertal hormonal milieu can influence the AN process, we have exploited two different mouse models characterized by alterations of HPG axis factors/secretions around puberty. Firstly, we studied the *GnRH::cre;Dicer*^*loxP*/*loxP*^ mice (Messina et al., 2016). These animals show a gradual loss of GnRH expression and secretion, which starts during the infantile period (p7-p20; Prevot, 2015) and accelerates in the juvenile animals after weaning (i.e., p21–~p35). Thus, in these mice the increased secretion of GnRH and downstream factors (LH, FSH, and gonadal hormones) driving the onset of puberty is impaired. This condition results in animals characterized by a severe hypogonadism and sterility strictly related to GnRH deficiency, as demonstrated by physiologic and anatomical analyses performed during adulthood (Messina et al., 2016). For example, a marked reduction of the serum level of gonadotropins (a proxy for GnRH secretion in smaller species) despite an intact pituitary function indicated that the hypogonadism and sterility in these animals were primarily due to a GnRH deficiency (Messina et al., 2016). Notably, the critical perinatal GnRH- independent (release of LH and testosterone) and -dependent organization of sex brain circuits remains preserved (O'Shaughnessy et al., 1998; Glanowska et al., 2014), indicating this model can be informative as concerns the impact of peri-pubertal alterations of HPG factors in shaping adult neural networks. In addition, to investigate among the HPG factors the relative contribution of gonadal hormones, we also analyzed mice gonadectomized just before the onset of puberty in which the level of gonadal hormones is depleted whereas GnRH and gonadotropins remain functionally active (Czieselsky et al., 2016; Dubois et al., 2016).

### HPG Axis Impairment in the *GnRH::cre;Dicer^*loxP*/*loxP*^* Mice Results in Peculiar Alterations of Adult Neurogenesis

The analysis of early steps of adult neurogenesis in *GnRH::cre;Dicer*^*loxP*/*loxP*^ mice showed a sex specific reduction in the number of proliferative progenitors (Ki67+ cells) and proliferating progenitors committed toward the neuronal lineage (Ki67+ and DCX+ cells) restricted to males and to the SVZ (Figure 1). In this neurogenic niche, this effect was limited only to the dorso-lateral subdomain, thus potentially involving only certain types of OB newborn interneuron progenitors (Merkle et al., 2007, 2014). Interestingly, a sexually dimorphic modulation of AN was also found when considering the process of newborn cell survival in the OB region, which shows a significant decrease in the survival of newly-generated cells selectively in the MOB of *GnRH::cre;Dicer*^*loxP*/*loxP*^ females (Figure 3). By contrast, both male and female *GnRH::cre;Dicer*^*loxP*/*loxP*^ mice do not show any alteration of progenitor proliferation, early neuronal specification, as well as newborn cell survival, in the DG. Overall, these data suggest that the HPG-axis secretory activity around puberty impacts the process of AN selectively in the SVZ neurogenic niche and that this activity appears critical to control sexually dimorphic aspects of neurogenesis during adulthood.

It is known that in rodents both level and type of circulating sexual hormones and expression of their receptors can differentially modulate the AN process in the two neurogenic niches, depending on a complex plethora of interrelated factors, which actually include sex, age, species, individual experience, physiologic and pathological states (Kuhn et al., 1996; Banasr et al., 2001; Leuner et al., 2007; Crews et al., 2010; Nunez-Parra et al., 2011; Epp et al., 2013; Díaz et al., 2017). Therefore, although the more mechanistic aspects underlying such sexually dimorphic and niche-specific alteration of AN in the *GnRH::cre;Dicer*^*loxP*/*loxP*^ mice need further investigation, the occurrence of functional impairment of the HPG axis affecting AN is rather expected. Nevertheless, one key point of this study arises by comparing the results (on both cell proliferation/differentiation and survival) obtained in *GnRH::cre;Dicer*^*loxP*/*loxP*^ mice (males and females), which are featured by a HPG impairment starting just before puberty, with those from models/studies wherein the impact of the HPG axis factors on AN has been evaluated during adulthood (i.e., in fully mature animals), or early postnatal life (i.e., in pre-/peri-pubertal animals; reviewed in Galea et al., 2013; Mahmoud et al., 2016).

For example, it is known that low level of circulating steroids, obtained through testicular removal during adulthood, affects cell survival in the DG of male rodents (Ormerod et al., 2004; Mak et al., 2007; Spritzer and Galea, 2007; Spritzer et al., 2011), whereas both cell proliferation and survival in the DG of *GnRH::cre;Dicer*^*loxP*/*loxP*^ males is not altered. Similarly, in females, endogenous fluctuations in ovarian hormones (Tanapat et al., 1999; Lagace et al., 2007; Rummel et al., 2010; Tzeng et al., 2014), aging (Barha et al., 2015), depletion (ovariectomy), and acute (Barker and Galea, 2008) but not chronic (Tanapat et al., 2005; Chan et al., 2014) replacement of estradiol, influence cell proliferation and survival in the DG, whereas proliferation and survival in the *GnRH::cre;Dicer*^*loxP*/*loxP*^ females are not affected in this region. Moreover, in the SVZ neurogenic niche, diverse HPG secreted factors, including gonadotropins, prolactin and estradiol can modulate cell proliferation during adulthood in female mice (Shingo et al., 2003; Mak et al., 2007; Brock et al., 2010; Larsen and Grattan, 2012), whereas the impairment of HPG activity does not modulate proliferation in *GnRH::cre;Dicer*^*loxP*/*loxP*^ females. In addition, gonadectomy in different strains of adult males increases the number of SVZ proliferating cells (Tatar et al., 2013), in opposition to what found in *GnRH::cre;Dicer*^*loxP*/*loxP*^ males. These data support the occurrence of a specific temporal modulatory relationship between the HPG factors and the process of adult neurogenesis.

Unique data in the *GnRH::cre;Dicer*^*loxP*/*loxP*^ mice arise also when it is considered the newborn cell survival in the OB, although a comparison with other models of HPG dysfunction in this region is complicated by the paucity of data available on this issue (see for review Ponti et al., 2018), and the fact that the number of newborn neurons integrating in the OBs depends on both the proliferation rate of SVZ progenitors (Larsen et al., 2008) and/or an activity-dependent survival (Oboti et al., 2011, 2017; Moreno et al., 2012; Oboti and Platel, 2012; Lepousez et al., 2014; Schellino et al., 2016). In the *GnRH::cre;Dicer*^*loxP*/*loxP*^ mice, a significant reduction of newborn cell survival was found in the MOB of females only (Figure 3). This result appears consistent with previous data showing that direct contact with pheromones, which actually stimulates the release of HPG axis factors (Gore et al., 2000; Richardson et al., 2004), promotes directly, or through increased SVZ proliferation, the survival of newborn neurons in the OB region of female mice (Shingo et al., 2003; Mak et al., 2007; Larsen et al., 2008; Oboti et al., 2009, 2011, 2017; Mak and Weiss, 2010; Larsen and Grattan, 2012; Schellino et al., 2016). Nevertheless, short-term treatment with estradiol reduces cell survival in the OB of adult female mice, as a consequence of a drop in SVZ proliferation (Brock et al., 2010). Moreover, exposure to estradiol in adult female-aromatase knockout mice, which are unable to produce estradiol across their entire lifespan (Bakker et al., 2002; Bakker, 2003), does not influence cell proliferation in the SVZ, but still reduces survival of newborn neurons in the MOB, but not in the AOB (Veyrac and Bakker, 2011; Brus et al., 2016). By contrast, the modulation of cell survival occurs in the AOB but not in the MOB when estradiol exposure starts before puberty (Veyrac and Bakker, 2011). Overall, besides the high level of complexity underlying the regulation of survival of newborn neurons via sexual hormones in the OB region, where newborn neurons are directly involved in the processing of salient cues (Mak et al., 2007; Larsen et al., 2008; Larsen and Grattan, 2010; Oboti et al., 2011; Schellino et al., 2016), these data support a diverse modulatory role of hormones depending on their exposure period (perinatal, pubertal or adult life).

### Cell Survival in the MOB Decreases in Pre-pubertal Ovariectomized Females

From the above discussion clearly emerges that the peculiar alterations of AN identified in the adult *GnRH::cre;Dicer*^*loxP*/*loxP*^ mice cannot be solely attributable to impaired secretion of sexual hormones during adulthood. To further interpret these results it is also necessary to consider that the inactivation of the GnRH peptide production in the *GnRH::cre;Dicer*^*loxP*/*loxP*^ mice results in impairment of secretory activity/factors along the whole HPG axis. Thus, it is likely that our data on AN arise from a crosstalk between direct and/or synergistic effect of each different HPG secretory factors (GnRH, gonadotropins, sex steroids and other circulating hormones). For example, as above mentioned, gonadotropins or prolactin alone, can actually modulate AN (Mak et al., 2007; Larsen et al., 2008; Larsen and Grattan, 2010). Moreover, previous studies on the GnRH peptide function indicate that GnRH *per se* may play extra-reproductive functions in the brain (Merchenthaler et al., 1989; Shinoda et al., 1989; Lin et al., 2004; Balasubramanian et al., 2010), and, accordingly, a link between GnRH secretion and DG neurogenesis has been previously suggested in aged mice (Zhang et al., 2013). To shed light on the relative contribution of diverse HPG axis factors in modulating AN at puberty, we chose to examine AN in females ovariectomized at p21 (soon before puberty) and treated with BrdU at p28, the puberty onset in mice (Oboti et al., 2017). Importantly, p28 it is exactly the age at which the GnRH peptide content significantly reduces in the *GnRH::cre;Dicer*^*loxP*/*loxP*^ mice (Messina et al., 2016). In the OVX model, instead of a general deficiency of the whole HPG axis secretory activity, we measured the effect given by the loss of (pre-pubertal) gonadal hormone secretion on AN. We focused on the survival of newborn neurons in the main OB of female animals, since it is significantly reduced in *GnRH::cre;Dicer*^*loxP*/*loxP*^ female mice. Our results on the pre-pubertal OVX females show the same trend found in the *GnRH::cre;Dicer*^*loxP*/*loxP*^ females (Figures 3, 6), although the ovariectomized mice were subjected to a hormonal depletion for a shorter period of time (i.e., 35 days) compared to the *GnRH::cre;Dicer*^*loxP*/*loxP*^ model (i.e., ~50 days) and despite AN occurs at different rates throughout life, with an age-related decline (Ben Abdallah et al., 2010; Spalding et al., 2013) that is sharper after puberty (He and Crews, 2007). This result suggests that the alterations of AN identified in this mutant model could be attributable to an impaired gonadal hormone secretion occurring during the peri-pubertal life, although we cannot exclude our results on OVX animals could be also influenced by GnRH or gonadotropins rise elicited in absence of the estrogen negative feedback on the GnRH system (Czieselsky et al., 2016; Dubois et al., 2016). Nevertheless, it is to note that a previous study in rodents (Farinetti et al., 2015) showed that impaired gonadal hormone secretion just before puberty (gonadectomy at p21), as in the *GnRH::cre;Dicer*^*loxP*/*loxP*^ male mice, results in decreased proliferation of primary and intermediate progenitors in a subregion of the SVZ neurogenic niche, which is restored by treatment with estradiol and testosterone, thus supporting our data regarding the involvement of peripubertal gonadal hormones in shaping the AN process.

In conclusion, this study, although not yet conclusive as regards the modulatory role on adult neurogenesis of each pre- and peri-pubertal secretion of the HPG-axis, underlines the pubertal activation of the HPG axis system (i.e., GnRH, gonadotropins, and gonadal hormones) is crucial to set up the AN process selectively in the SVZ. Interestingly, this mechanism seems to occur differentially in males and females, influencing cell proliferation in males and cell survival in females, thus suggesting that it is critical for establishing a sexually dimorphic function of the AN process.

## Data Availability Statement

The raw data supporting the conclusions of this article will be made available by the authors, without undue reservation.

## Ethics Statement

The animal study was reviewed and approved by Institutional Ethics Committees for the Care and Use of Experimental Animals of the Universities of Lille (APAFIS#13387-2017122712209790 v9) and Turin (Protocol Number DGSAF0007085-A05/04/2013).

## Author Contributions

ST performed the experiments, analyzed the data, and revised the manuscript. SBov analyzed the data, interpreted the results, and revised the manuscript. GP performed experiments. SBon analyzed the data and revised the manuscript. PG interpreted the results and revised the manuscript. PP interpreted the results and wrote the manuscript. All authors contributed to the article and approved the submitted version.

## Conflict of Interest

The authors declare that the research was conducted in the absence of any commercial or financial relationships that could be construed as a potential conflict of interest.
