# Supplementary material for: HPG-Dependent Peri-Pubertal Regulation of Adult Neurogenesis in Mice
Source: Front Neuroanat. 2020 Nov 27;14:584493. doi: 10.3389/fnana.2020.584493 (PMC7732626; doi:10.3389/fnana.2020.584493)
Supplement: Supplementary file 1 [file Data_Sheet_1.PDF]

Supplementary Material

1 Supplementary Figures

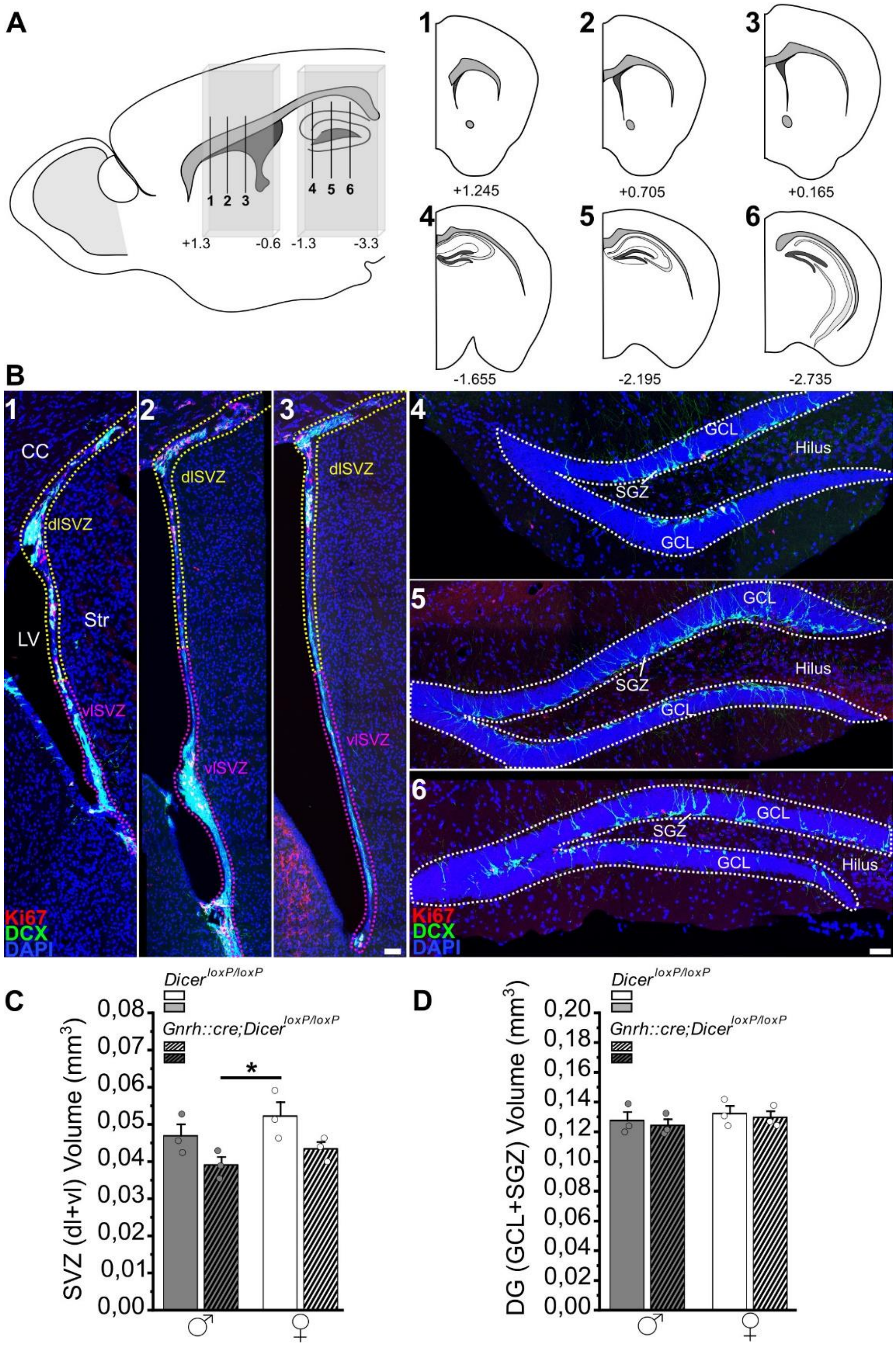

Supplementary Figure 1. A. Schematic of the sagittal view of a mouse brain slice at the level of the SVZ and Hippocampus (upper left). Grey solids represent the region of the SVZ and DG and relatives Bregma coordinates, whose volumes are reported in panels C and D. For each region, three slices (numbered 1 to 3 for the SVZ and 4 to 6 for the DG) positioned at different Bregma coordinates (upper right) have been considered for cell counts. **B.** Representative coronal sections of the SVZ (left) and DG (right) immunolabeled for Ki67 (red), DCX (green) and DAPI (blue). Scale bar in B3 and B6 = 50 µm. **C-D.** Volumes of SVZdl+vl (C) and GCL+SGZ (D) in *Dicer*$^{loxP/loxP}$ and *GnRH::cre/Dicer*$^{loxP/loxP}$ male (n=3) and female (n=3) mice. Two-way ANOVA and Tukey post-test. *p = 0.042. vlSVZ, ventro-lateral subventricular zone; dlSVZ, dorso-lateral subventricular zone; CC, corpus callosum; Str, striatum; LV, lateral ventricle; GCL, granule cell layer; SGZ, subgranular zone.

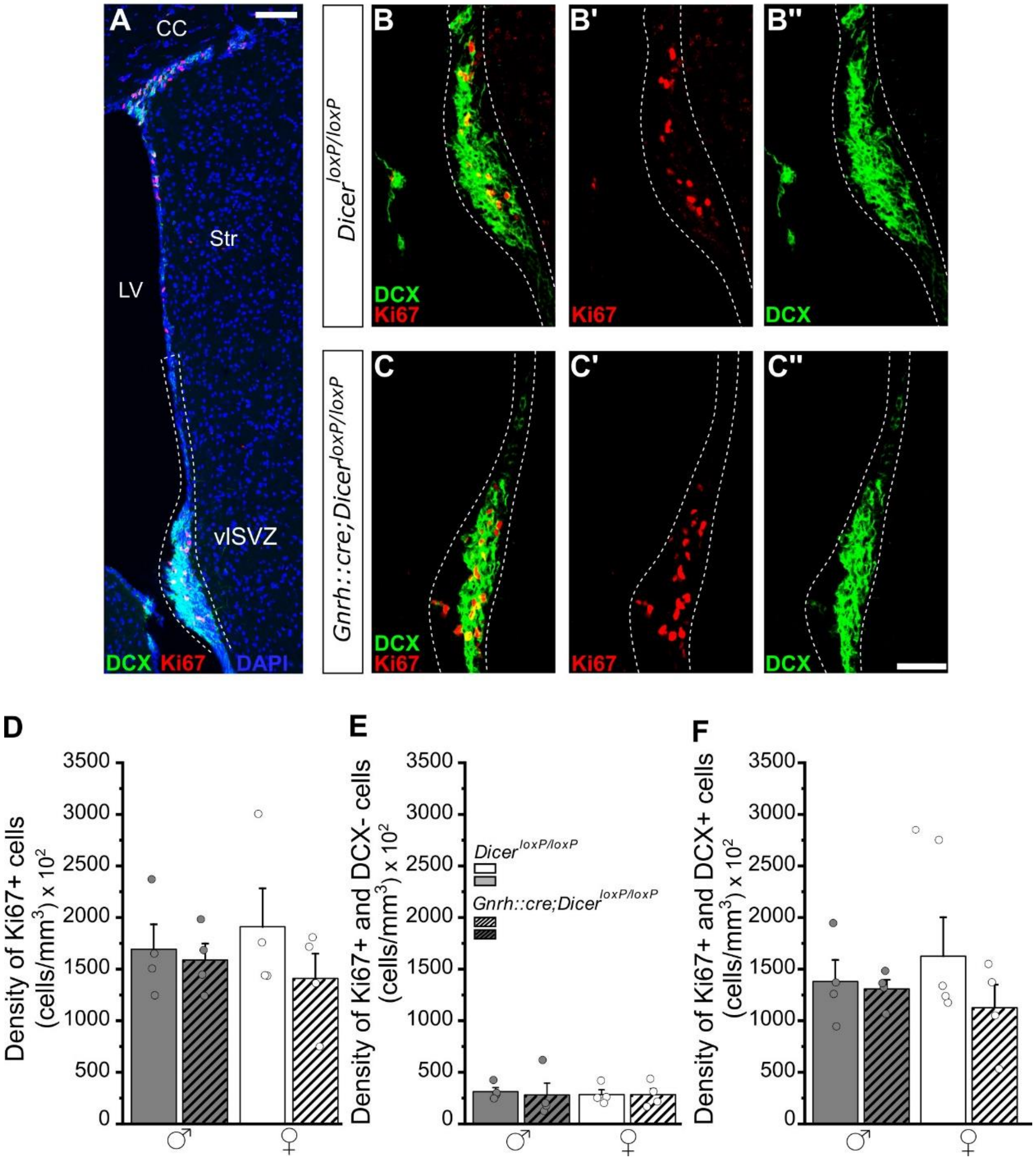

Supplementary Figure 2. Progenitor proliferation and neuronal differentiation in the ventro-lateral SVZ (vlSVZ) of *GnRH::cre/Dicer*$^{loxP/loxP}$ and *Dicer*$^{loxP/loxP}$ mice. A. Representative coronal section of the SVZ, immunolabeled for Ki67 (red), DCX (green) and DAPI (blue) in an adult (P90) *Dicer*$^{loxP/loxP}$ male mouse. The dotted line indicates the vlSVZ. Scale bar in A = 100 µm. **B-C.** Immunofluorescence for DCX and Ki67 (B and C), for Ki67 only (B' and C') and for DCX only (B'' and C'') at the level of the vlSVZ in *Dicer*$^{loxP/loxP}$ (B-B'') and *GnRH::cre/Dicer*$^{loxP/loxP}$ male mice (C-C''). Scale bar in C''= 50 µm and applies to B-C, B'-C' and B''. **D-F.** Density of Ki67+ (D), Ki67+/DCX- (E) and Ki67+/DCX+ (F) cells in the vlSVZ of *Dicer*$^{loxP/loxP}$ and *GnRH::cre/Dicer*$^{loxP/loxP}$ male (n=4) and female mice (n=4). Two-way ANOVA and Tukey post-test. vlSVZ, ventro-lateral subventricular zone; CC, corpus callosum; Str, striatum; LV, lateral ventricle.

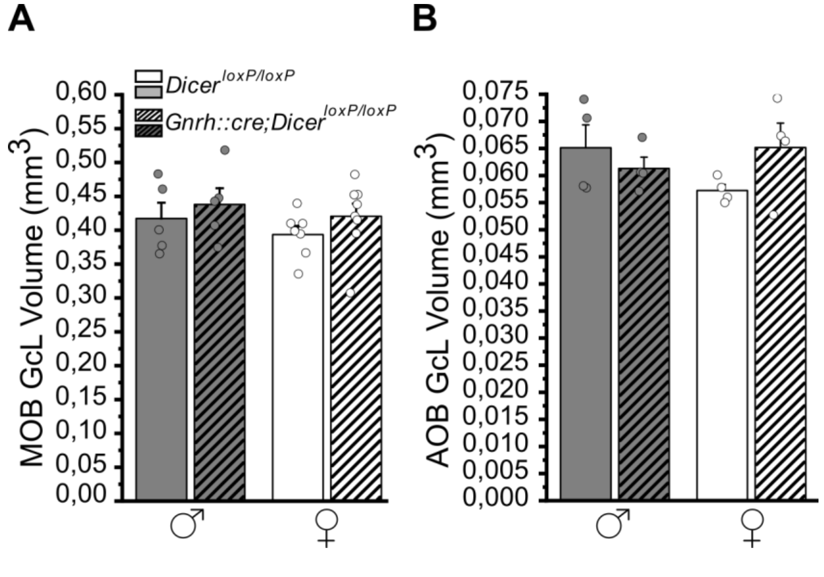

Supplementary Figure 3. Volumes of MOB GcL in *Dicer*$^{loxP/loxP}$ and *GnRH::cre/Dicer*$^{loxP/loxP}$ male (n=5) and female (n=7 control and n=8 *GnRH::cre/Dicer*$^{loxP/loxP}$) mice. Two-way ANOVA and Tukey post-test. **B.** Volumes of AOB GcL in *Dicer*$^{loxP/loxP}$ and *GnRH::cre/Dicer*$^{loxP/loxP}$ male (n=4) and female (n=4) mice. Two-way ANOVA and Tukey post-test.
