# Supplementary material for: HPG-Dependent Peri-Pubertal Regulation of Adult Neurogenesis in Mice
Source: Front Neuroanat. 2020 Nov 27;14:584493. doi: 10.3389/fnana.2020.584493 (PMC7732626; doi:10.3389/fnana.2020.584493)
Supplement: Supplementary file 2 [file Data_Sheet_2.PDF]

Supplementary Material

1. Supplementary Table 1.1

Ki67+ cell density (cells/mm^3) in the dlSVZ					
Two-way ANOVA	Ordinary				
Alpha	0,05				
Source of Variation	% of total variation	P value	P value	Significant?	
Interaction	20,28	0,0331	*	Yes	
Gender	0,1421	0,8437	ns	No	
Genotype	37,57	0,0066	**	Yes	
ANOVA table	SS	DF	MS	F (DFn. DFd)	P value
Interaction	8,78924E9	1	8,78924E9	F (1, 12) = 5,795	P=0,0331
Gender	6,15517E7	1	6,15517E7	F (1, 12) = 0,0406	P=0,8437
Genotype	1,62793E10	1	1,62793E10	F (1, 12) = 10,733	P=0,0066
Residual	1,82002E10	12	1,51668E9		

**Tukey's multiple comparisons test**	**MeanDiff**	**95.00% CI of diff.**	**Summary**	**P value**
m wt vs. m ko	110670,63	28913,27 to 192428,0	**	0,0080
m wt vs. f wt	42952,74	-38804,63 to 124710,1	ns	0,4353
m wt vs. f ko	59872,41	-21884,95 to 141629,78	ns	0,1857
m ko vs. f wt	-67717,90	-149475,27 to 14039,47	ns	0,1181
m ko vs. f ko	-50798,22	-132555,59 to 30959,14	ns	0,3008
f wt vs. f ko	16919,68	-64837,69 to 98677,04	ns	0,9255

2. Supplementary Table 1.2

Ki67+ and DCX- cell density (cells/mm^3) in the dlSVZ					
Two-way ANOVA	Ordinary				
Alpha	0,05				
Source of Variation	% of total variation	P value	P value	Significant?	
Interaction	4,299	0,3944	ns	No	
Gender	10,34	0,1958	ns	No	
Genotype	19,25	0,0862	ns	No	
ANOVA table	SS	DF	MS	F (DFn. DFd)	P value
Interaction	1,73893E8	1	1,73893E8	F (1, 12) = 0,7804	P=0,3944
Gender	4,18162E8	1	4,18162E8	F (1, 12) = 1,877	P=0,1958
Genotype	7,78666E8	1	7,78666E8	F (1, 12) = 3,494	P=0,0862
Residual	2,67398E9	12	2,22832E8		

**Tukey's multiple comparisons test**	**MeanDiff**	**95.00% CI of diff.**	**Summary**	**P value**
m wt vs. m ko	20545,7264	-10792,04 to 51883,50	ns	0,2606
m wt vs. f wt	-3631,07703	-34968,85 to 27706,69	ns	0,9853
m wt vs. f ko	3727,7928	-27609,98 to 35065,56	ns	0,9842
m ko vs. f wt	-24176,8034	-55514,57 to 7160,97	ns	0,1549
m ko vs. f ko	-16817,9336	-48155,70 to 14519,84	ns	0,4179
f wt vs. f ko	7358,8698	-23978,90 to 38696,64	ns	0,8962

3. Supplementary Table 1.3

**Ki67+ and DCX+ cell density (cells/mm^3) in the dlSVZ**					
Two-way ANOVA	Ordinary				
Alpha	0,05				
Source of Variation	% of total variation	P value	P value	Significant?	
Interaction	22,25	0,0285	*	Yes	
Gender	0,5445	0,7040	ns	No	
Genotype	34,07	0,0096	**	Yes	
ANOVA table	SS	DF	MS	F (DFn. DFd)	P value
Interaction	6,49057E9	1	6,49057E9	F (1, 12) = 6,189	P=0,0285
Gender	1,58849E8	1	1,58849E8	F (1, 12) = 0,1515	P=0,7040
Genotype	9,93724E9	1	9,93724E9	F (1, 12) = 9,476	P=0,0096
Residual	11326040	12	943837		

**Tukey's multiple comparisons test**	**MeanDiff**	**95.00% CI of diff.**	**Summary**	**P value**
m wt vs. m ko	90124,9098	22140,88 to 158108,93	**	0,0092
m wt vs. f wt	46583,8132	-21400,21 to 114567,84	ns	0,2293
m wt vs. f ko	56144,6189	-11839,41 to 124128,64	ns	0,1195
m ko vs. f wt	-43541,0967	-111525,12 to 24442,93	ns	0,2779
m ko vs. f ko	-33980,2909	-101964,32 to 34003,73	ns	0,4759
f wt vs. f ko	9560,8058	-58423,22 to 77544,83	ns	0,9744

4. Supplementary Table 1.4

Ki67+ cell density (cells/mm^3) in the vlSVZ					
Two-way ANOVA	Ordinary				
Alpha	0,05				
Source of Variation	% of total variation	P value	P value	Significant?	
Interaction	4,031	0,4690	ns	No	
gender	0,03611	0,9447	ns	No	
genotype	9,422	0,2753	ns	No	
ANOVA table	SS	DF	MS	F (DFn. DFd)	P value
Interaction	1,56592E9	1	1,56592E9	F (1, 12) = 0,5591	P=0,4690
gender	1,40272E7	1	1,40272E7	F (1, 12) = 0,0050	P=0,9447
genotype	3,6602E9	1	3,6602E9	F (1, 12) = 1,307	P=0,2753
Residual	3,36091E10	12	2,80076E9		

**Tukey's multiple comparisons test**	**MeanDiff**	**95.00% CI of diff.**	**Summary**	**P value**
m wt vs. m ko	10463,9439	-100636,89 to 121564,77	ns	0,9920
m wt vs.f wt	-21658,48177	-132759,31 to 89442,35	ns	0,9366
m wt vs.f ko	28377,12892	-82723,70 to 139477,96	ns	0,8714
m ko vs.f wt	-32122,42567	-143223,26 to 78978,41	ns	0,8257
m ko vs.f ko	17913,18502	-93187,65 to 129014,02	ns	0,9624
f wt vs.f ko	50035,61069	-61065,22 to 161136,44	ns	0,5586

5. Supplementary Table 1.5

Ki67+ and DCX- cell density (cells/mm^3) in the vlSVZ					
Two-way ANOVA	Ordinary				
Alpha	0,05				
Source of Variation	% of total variation	P value	P value	Significant?	
Interaction	0,4185	0,8254	ns	No	
gender	0,2966	0,8527	ns	No	
genotype	0,45	0,8191	ns	No	
ANOVA table	SS	DF	MS	F (DFn. DFd)	P value
Interaction	1,02939E7	1	1,02939E7	F (1, 12) = 0,0508	P=0,8254
gender	7,29571E6	1	7,29571E6	F (1, 12) = 0,0360	P=0,8527
genotype	1,1068E7	1	1,1068E7	F (1, 12) = 0,0546	P=0,8191
Residual	2,431E9	12	2,02583E8		

Tukey’s multiple comparisons test	MeanDiff	95.00% CI of diff.	Summary	P value
m wt vs. m ko	3267,6393	-26612,43 to 33147,71	ns	0,9876
m wt vs.f wt	2954,7367	-26925,34 to 32834,81	ns	0,9907
m wt vs.f ko	3013,9589	-26866,11 to 32894,03	ns	0,9902
m ko vs.f wt	-312,9025	-30192,98 to 29567,17	ns	>0,9999
m ko vs.f ko	-253,6803	-30133,76 to 29626,39	ns	>0,9999
f wt vs.f ko	59,2222	-29820,85 to 29939,30	ns	>0,9999

6. Supplementary Table 1.6

Ki67+ and DCX+ cell density (cells/mm^3) in the vlSVZ					
Two-way ANOVA	Ordinary				
Alpha	0,05				
Source of Variation	% of total variation	P value	P value	Significant?	
Interaction	5,33	0,4028	ns	No	
gender	0,121	0,8982	ns	No	
genotype	9,519	0,2690	ns	No	
ANOVA table	SS	DF	MS	F (DFn. DFd)	P value
Interaction	1,83014E9	1	1,83014E9	F (1, 12) = 0,7522	P=0,4028
gender	4,15555E7	1	4,15555E7	F (1, 12) = 0,0171	P=0,8982
genotype	3,26872E9	1	3,26872E9	F (1, 12) = 1,343	P=0,2690
Residual	2,91977E10	12	2,43314E9		

Tukey’s multiple comparisons test	MeanDiff	95.00% CI of diff.	Summary	P value
m wt vs. m ko	7196,30463	-96356,79 to 110749,40	ns	0,9967
m wt vs. f wt	-24613,2185	-128166,32 to 78939,88	ns	0,8929
m wt vs. f ko	25363,16999	78189,93 to 128916,27	ns	0,8844
m ko vs. f wt	-31809,5231	-135362,62 to 71743,57	ns	0,7990
m ko vs. f ko	18166,86536	-85386,23 to 121719,96	ns	0,9524
f wt vs. f ko	49976,38849	-53576,71 to 153529,49	ns	0,5042

7. Supplementary Table 1.7

SVZ volume (mm^3)					
Two-way ANOVA	Ordinary				
Alpha	0,05				
Source of Variation	% of total variation	P value	P value	Significant?	
Interaction	0,1482	0,8681	ns	No	
gender	14,97	0,1232	ns	No	
genotype	44,55	0,0178	*	Yes	
ANOVA table	SS	DF	MS	F (DFn. DFd)	P value
Interaction	6,928e-007	1	6,928e-007	F (1, 8) = 0,0294	P=0,8681
gender	6,997e-005	1	6,997e-005	F (1, 8) = 2,969	P=0,1232
genotype	0,0002082	1	0,0002082	F (1, 8) = 8,836	P=0,0178
Residual	0,0001885	8	2,356e-005		

Tukey's multiple comparisons test	MeanDiff	95.00% CI of diff.	Summary	P value
m ko vs. m wt	-0,00785	-0,0205 to 0,004842	ns	0,2707
f wt vs. m wt	0,00531	-0,007383 to 0,018	ns	0,5658
f wt vs. m ko	0,01316	-0,0005 to 0,02585	*	0,0424
f ko vs. m wt	-0,0035	-0,0162 to 0,00919	ns	0,8135
f ko vs. m ko	0,00435	-0,00834 to 0,01704	ns	0,7010
f ko vs. f wt	-0,00881	-0,0215 to 0,003881	ns	0,1965

8. Supplementary Table 2.1

Ki67+ and DCX- cell density (cells/mm^3) in the DG					
Two-way ANOVA	Ordinary				
Alpha	0,05				
Source of Variation	% of total variation	P value	P value	Significant?	
Interaction	0,8619	0,7083	ns	No	
gender	3,123	0,4795	ns	No	
genotype	17,83	0,1055	ns	No	
ANOVA table	SS	DF	MS	F (DFn. DFd)	P value
Interaction	88481,87775	1	88481,88	F (1, 13) = 0,1463	P=0,7083
gender	320637,4828	1	320637,49	F (1, 13) = 0,53	P=0,4795
genotype	1,83073E6	1	1,83073E6	F (1, 13) = 3,026	P=0,1055
Residual	7,86402E6	13	604924,822		

Tukey's multiple comparisons test	MeanDiff	95.00% CI of diff.	Summary	P value
m ko vs. m wt	-514,42936	-2128,63 to 1099,77	ns	0,7868
f wt vs. m wt	-130,99183	-1745,19 to 1483,21	ns	0,9950
f wt vs. m ko	383,43753	-1230,76 to 1997,64	ns	0,8963
f ko vs. m wt	-935,3484	-2466,71 to 596,02	ns	0,3198
f ko vs. m ko	-420,9190	-1952,29 to 1110,45	ns	0,8501
f ko vs. f wt	-804,35657	-2335,72 to 727,01	ns	0,4426

9. Supplementary Table 2.2

	Ki67+ and DCX+ cell density (cells/mm^3) in the DG				
Two-way ANOVA	Ordinary				
Alpha	0,05				
Source of Variation	% of total variation	P value	P value	Significant?	
Interaction	3,846	0,4697	ns	No	
gender	2,413	0,5654	ns	No	
genotype	3,969	0,4628	ns	No	
ANOVA table	SS	DF	MS	F (DFn. DFd)	P value
Interaction	1,65828E6	1	1,65828E6	F (1, 13) = 0,5546	P=0,4697
gender	1,04053E6	1	1,04053E6	F (1, 13) = 0,348	P=0,5654
genotype	1,71137E6	1	1,71137E6	F (1, 13) = 0,5723	P=0,4628
Residual	3,88726E7	13	2,9902E6		

Tukey's multiple comparisons test	MeanDiff	95.00% CI of diff.	Summary	P value
m ko vs. m wt	-1265,10109	-4853,97 to 2323,77	ns	0,7329
f wt vs. m wt	-130,45076	-3719,32 to 3458,42	ns	0,9995
f wt vs. m ko	1134,65033	-2454,22 to 4723,52	ns	0,7907
f ko vs. m wt	-140,41728	-3545,12 to 3264,28	ns	0,9993
f ko vs. m ko	1124,68381	-2280,02 to 4529,38	ns	0,7686
f ko vs. f wt	-9,96652	-3414,67 to 3394,73	ns	>0,9999

10. Supplementary Table 2.3

DCX+ and Ki67- cell density (cells/mm³) in the DG					
Two-way ANOVA	Ordinary				
Alpha	0,05				
Source of Variation	% of total variation	P value	P value	Significant?	
Interaction	1,171	0,5695	ns	No	
gender	41,73	0,0040	**	Yes	
genotype	15,17	0,0557	ns	No	
ANOVA table	SS	DF	MS	F (DFn. DFd)	P value
Interaction	3,97785E7	1	3,97785E7	F (1, 13) = 0,3405	P=0,5695
gender	1,41771E9	1	1,41771E9	F (1, 13) = 12,139	P=0,0040
genotype	5,15527E8	1	5,15527E8	F (1, 13) = 4,4127	P=0,0557
Residual	1,51877E9	13	1,16828E8		

Tukey's multiple comparisons test	MeanDiff	95.00% CI of diff.	Summary	P value
m ko vs. m wt	-7991,4957	-30424,19 to 14441,20	ns	0,7267
f wt vs. m wt	21423,2090	-1009,48 to 43855,90	ns	0,0632
f wt vs. m ko	29414,7047	6982,01 to 51847,40	**	0,0095
f ko vs. m wt	7284,38965	-13997,13 to 28565,91	ns	0,7495
f ko vs. m ko	15275,8853	-6005,64 to 36557,41	ns	0,2019
f ko vs. f wt	-14138,8194	-35420,34 to 7142,70	ns	0,2557

11. Supplementary Table 2.4

DG volume (mm³)					
Two-way ANOVA	Ordinary				
Alpha	0,05				
Source of Variation	% of total variation	P value	P value	Significant?	
Interaction	0,06048	0,9416	ns	No	
gender	11,32	0,3317	ns	No	
genotype	3,825	0,5646	ns	No	
ANOVA table	SS	DF	MS	F (DFn. DFd)	P value
Interaction	4,027e-007	1	4,027e-007	F (1, 8) = 0,00571	P=0,9416
gender	7,535e-005	1	7,535e-005	F (1, 8) = 1,068	P=0,3317
genotype	2,547e-005	1	2,547e-005	F (1, 8) = 0,3609	P=0,5646
Residual	0,0005646	8	7,057e-005		

Tukey's multiple comparisons test	MeanDiff	95.00% CI of diff.	Summary	P value
m wt vs. m ko	-0,00328	-0,02525 to 0,01869	ns	0,9618
f ko vs. m ko	0,004645	-0,01732 to 0,02661	ns	0,9029
f ko vs. m wt	0,007925	-0,01404 to 0,02989	ns	0,6686
f wt vs. m ko	0,002098	-0,01987 to 0,02406	ns	0,9893
f wt vs. m wt	0,005378	-0,01659 to 0,02734	ns	0,8597
f wt vs. f ko	-0,002547	-0,02451 to 0,01942	ns	0,9813

12. Supplementary Table 3.1

GcL BrdU cell density (cells/mm^3) in the MOB					
Two-way ANOVA	Ordinary				
Alpha	0,05				
Source of Variation	% of total variation	P value	P value	Significant?	
Interaction	22,3	0,0094	**	Yes	
Gender	10,01	0,0693	ns	No	
Genotype	5,875	0,1574	ns	No	
ANOVA table	SS	DF	MS	F (DFn. DFd)	P value
Interaction	98645414	1	98645414	F (1, 21) = 8,16	P=0,0094
Gender	44286084	1	44286084	F (1, 21) = 3,663	P=0,0693
Genotype	25989310	1	25989310	F (1, 21) = 2,15	P=0,1574
Residual	253862594	21	12088695		

Tukey's multiple comparisons test	MeanDiff	95.00% CI of diff.	Summary	P value
m wt vs. m ko	-1975,2615	-8104,45 to 4153,92	ns	0,8058
m wt vs. f wt	-6777,58375	-12452,11 to -1103,06	*	0,0156
m wt vs. f ko	-636,13135	-6160,90 to 4888,64	ns	0,9882
m ko vs. f wt	-4802,32224	-10477,84 to 872,20	ns	0,1166
m ko vs. f ko	1339,13016	-4186,64 to 6863,90	ns	0,9052
f wt vs. f ko	6141,4524	1126,83 to 11157,07	*	0,0129

13. Supplementary Table 3.2

GcL MOB volume (mm^3)					
Two-way ANOVA	Ordinary				
Alpha	0,05				
Source of Variation	% of total variation	P value	P value	Significant?	
Interaction	0,1081	0.9670	ns	No	
Gender	4,652	0,2989	ns	No	
Genotype	6,201	0,2692	ns	No	
ANOVA table	SS	DF	MS	F (DFn. DFd)	P value
Interaction	4,16758E-6	1	4,16758E-6	F (1, 21) = 0,0017	P= 0,9670
Gender	0,0027	1	0,00307	F (1, 21) = 1,2913	P= 0,2692
Genotype	0,00307	1	0,0027	F (1, 21) = 1,1374	P= 0,2989
Residual	0,04872	21	0,00232		

Tukey's multiple comparisons test	MeanDiff	95.00% CI of diff.	Summary	P value
m wt vs. m ko	-0,02068	-0,10698 to 0,06562	ns	0,9069
m wt vs. f wt	0,02378	-0,05612 to 0,10368	ns	0,83812
m wt vs. f ko	0,00141	-0,07849 to 0,08131	ns	0,99995
m ko vs. f wt	0,04447	-0,03543 to 0,12437	ns	0,42391
m ko vs. f ko	0,02209	-0,05781 to 0,10199	ns	0,86527
f wt vs. f ko	-0,02237	-0,09531 to 0,05057	ns	0,82582

14. Supplementary Table 4.1

GcL BrdU cell density (cells/mm^3) in the AOB					
Two-way ANOVA	Ordinary				
Alpha	0,05				
Source of Variation	% of total variation	P value	P value	Significant?	
Interaction	7,776	0,2751	ns	No	
Gender	18,16	0,1061	ns	No	
Genotype	2,709	0,5125	ns	No	
ANOVA table	SS	DF	MS	F (DFn. DFd)	P value
Interaction	2723058	1	2723058	F (1, 12) = 1,308	P=0,2751
Gender	6358696	1	6358696	F (1, 12) = 3,053	P=0,1061
Genotype	948620	1	948620	F (1, 12) = 0,455	P=0,5125
Residual	24989308	12	2082442		

Tukey's multiple comparisons test	MeanDiff	95.00% CI of diff.	Summary	P value
m wt vs. m ko	-338,10	-3367,57 to 2691,37	ns	0,9868
m wt vs. f wt	-2085,91	-5115,37 to 943,56	ns	0,2259
m wt vs. f ko	-773,84	-3803,30 to 2256,63	ns	0,8714
m ko vs. f wt	-1747,81	-4777,27 to 1281,66	ns	0,3592
m ko vs. f ko	-435,74	-3465,20 to 2593,73	ns	0,9727
f wt vs. f ko	1312,07	-1717,39 to 4341,54	ns	0,5883

15. Supplementary Table 4.2

GcL BrdU cell density (cells/mm^3) in the aAOB					
Two-way ANOVA	Ordinary				
Alpha	0,05				
Source of Variation	% of total variation	P value	P value	Significant?	
Interaction	4,971	0,3369	ns	No	
Gender	27,31	0,0371	*	Yes	
Genotype	8,118	0,2252	ns	No	
ANOVA table	SS	DF	MS	F (DFn. DFd)	P value
Interaction	2341385	1	2341385	F (1, 12) = 1,001	P=0,3369
Gender	12863701	1	12863701	F (1, 12) = 5,499	P=0,0371
Genotype	3824028	1	3824028	F (1, 12) = 1,635	P=0,2252
Residual	28073844	12	2339487		

Tukey's multiple comparisons test	MeanDiff	95.00% CI of diff.	Summary	P value
m wt vs. m ko	212,67	-2998,32 to 3424,67	ns	0,9972
m wt vs. f wt	-2558,38	-5769,38 to 652,62	ns	0,1374
m wt vs. f ko	-815,54	-4027,54 to 2395,45	ns	0,8732
m ko vs. f wt	-2771,06	-5982,05 to 439,94	ns	0,0997
m ko vs. f ko	-1028,22	-4239,22 to 2182,77	ns	0,7788
f wt vs. f ko	1743,83	-1468,16 to 4953,83	ns	0,4087

16. Supplementary Table 4.3

GcL BrdU cell density (cells/mm^3) in the pAOB					
Two-way ANOVA	Ordinary				
Alpha	0,05				
Source of Variation	% of total variation	P value	P value	Significant?	
Interaction	10,08	0,2510	ns	No	
Gender	6,823	0,3405	ns	No	
Genotype	0,0001843	0,9960	ns	No	
ANOVA table	SS	DF	MS	F (DFn. DFd)	P value
Interaction	3133537	1	3133537	F (1, 12) = 1,455	P=0,2510
Gender	2121948	1	2121948	F (1, 12) = 0,9852	P=0,3405
Genotype	57,30414	1	57,3	F (1, 12) = 2,661e-005	P=0,9960
Residual	25845220	12	2153768		

Tukey's multiple comparisons test	MeanDiff	95.00% CI of diff.	Summary	P value
m wt vs. m ko	-888,87	-3969,79 to 2192,04	ns	0,8266
m wt vs. f wt	-1613,43	-4694,35 to 1467,48	ns	0,4379
m wt vs. f ko	-732,113	-3813,04 to 2348,78	ns	0,8930
m ko vs. f wt	-724,56	-3805,47 to 2356,35	ns	0,8958
m ko vs. f ko	156,74	-2924,17 to 3237,66	ns	0,9987
f wt vs. f ko	881,30	-2199,61 to 3962,22	ns	0,8301

17. Supplementary Table 4.4

GcL AOB volume (mm^3)					
Two-way ANOVA	Ordinary				
Alpha	0,05				
Source of Variation	% of total variation	P value	P value	Significant?	
Interaction	19,86	0,1007	ns	No	
Gender	2,338	0,5531	ns	No	
Genotype	2,448	0,5441	ns	No	
ANOVA table	SS	DF	MS	F (DFn. DFd)	P value
Interaction	1,38327E14	1	1,38327E14	F (1,12) = 3,1621	P=0,1007
Gender	1,62865E13	1	1,62865E13	F (1,12) = 0,3723	P=0,5531
Genotype	1,70557E13	1	1,70557E13	F (1,12) = 0,3899	P=0,5441
Residual	5,24939E14	12	4,37449E13		

Tukey's multiple comparisons test	MeanDiff	95.00% CI of diff.	Summary	P value
m wt vs. m ko	3,81569E6	-1,00692E7 to 1,77006E7	ns	0,8459
m wt vs. f wt	7,89845E6	-5,98648E6 to 2,17834E7	ns	0,3706
m wt vs. f ko	-47098,6875	-1,3932E7 to 1,38378E7	ns	>0,9999
m ko vs. f wt	4,08275E6	-9,80217E6 to 1,79677E7	ns	0,8186
m ko vs. f ko	-3,86279E6	-1,77477E7 to 1,00221E7	ns	0,8412
f wt vs. f ko	-7,94554E6	-2,18305E7 to 5,93938E6	ns	0,3658

18. Supplementary Table 5.1

GCL BrdU cell density (cells/mm^3) in the DG					
Two-way ANOVA	Ordinary				
Alpha	0,05				
Source of Variation	% of total variation	P value	P value	Significant?	
Interaction	0,6989	0,6989	ns	No	
Gender	0,2468	0,2468	ns	No	
Genotype	0,1025	0,1025	ns	No	
ANOVA table	SS	DF	MS	F (DFn. DFd)	P value
Interaction	186211	1	186211	F (1,22) = 0,1535	P = 0,6989
Gender	1,72E+06	1	1717005	F (1,22) = 1,416	P = 0,2468
Genotype	3,52E+06	1	3521898	F (1,22) = 2,904	P = 0,1025
Residual	2,67E+07	22	1,21E+06		

Tukey's multiple comparisons test	MeanDiff	95.00% CI of diff.	Summary	P value
m ko vs. m wt	-589,98025	-2563,93103 to 1383,97053	ns	0,8397
f wt vs. m wt	711,11413	-1161,54001 to 2583,76826	ns	0,7199
f wt vs. m ko	1301,09438	-350,43134 to 2952,62009	ns	0,1579
f ko vs. m wt	-231,20169	-2103,85583 to 1641,45245	ns	0,9858
f ko vs. m ko	358,77856	-1292,74715 to 2010,30428	ns	0,9299
f ko vs. f wt	-942,31581	-2471,33151 to 586,69989	ns	0,3419

19. Supplementary Table 5.2

GCL DG volume (mm^3)					
Two-way ANOVA	Ordinary				
Alpha	0,05				
Source of Variation	% of total variation	P value	P value	Significant?	
Interaction	4,393	0,2896	ns	No	
Gender	0,1718	0,8318	ns	No	
Genotype	16,72	0,0457	*	Yes	
ANOVA table	SS	DF	MS	F (DFn, DFd)	P value
Interaction	4,01123E-4	1	4,01123E-4	F (1,24) = 1,177	P=0,2896
Gender	1,57256E-5	1	1,57256E-5	F (1,24) = 0,046	P=0,8318
Genotype	0,00153	1	0,00153	F (1,24) = 4,484	P=0,0457
Residual	0,00749	22	3.40605E-4		

Tukey's multiple comparisons test	MeanDiff	95.00% CI of diff.	Summary	P value
m ko vs. m wt	-0,02413	-0.05721 to 0.00895	ns	0,20914
f wt vs. m wt	-0,0098	-0.04118 to 0.02159	ns	0,82178
f wt vs. m ko	0,01434	-0.01334 to 0.04201	ns	0,48994
f ko vs. m wt	-0,01757	-0.04896 to 0.01381	ns	0,42357
f ko vs. m ko	0,00656	-0.02112 to 0.03423	ns	0,91163
f ko vs. f wt	-0,00778	-0.0334 to 0.01784	ns	0,83342

20. Supplementary Table 6.1

GcL BrdU cell density (cells/mm3) in the MOB of OVX-SHAM females					
Two sample *t* Test					
Alpha	0,05				
Descriptive Statistics	N	Mean	SD	SEM	Median
	6	16114,43167	3815,3023	1557,59	15334,11
	5	11663,308	797,31602	356,5705	11542,47
Difference		4451,12367		1751,804	
Overall	11	14091,19364	3596,6596	1084,434	12443,79
t-Test Statistics	t Statistics	DF	P value		
Sham vs. OVX	2,54088	9	0,03167		

21. Supplementary Table 6.2

GcL Volume (mm^3) in the MOB of OVX-SHAM females					
Two sample *t* Test					
Alpha	0,05				
Descriptive Statistics	N	Mean	SD	SEM	Median
	6	0,61006	0,04314	0,01761	0,5925
	5	0,60047	0,06924	0,03097	0,59628
Difference		0,00959		0,03407	
Overall	11	0,6057	0,05361	0,01616	0,59628
t-Test Statistics	t Statistics	DF	P value		
Sham vs. OVX	0,28141	9	0,78476		

22. Supplementary Table 7

Primers used for genotyping		
**Primer**	**Sequence 5' → 3'**	**Genotype**
D-F1	CCT GAC AGT GAC GGT CCA AAG	*Dicer*$^{loxP/loxP}$
D-R1	CAT GAC TCT TCA ACT CAA ACT	
gCre-F1	CTG GTG TAG CTG ATG ATC CG	*Gnrh*::Cre
gCre-F2	ATG GCT AAT CGC CAT CTT CC	
